# SparseTrack: A Physics-Informed Transformer Framework for Real-Time Human Motion Reconstruction from Sparse IMUs

**DOI:** 10.3390/s26103262

**Published:** 2026-05-21

**Authors:** Adithya Balasubramanyam, Suchir Murali Velpanur, Sushma Edhala Jeevarathnam, Tejasree Chekuri Jayachandra, Prasad Honnavalli, Gowri Srinivasa

**Affiliations:** Department of Computer Science and Engineering, PES University, Bengaluru 560085, India; smv1524@gmail.com (S.M.V.); sushmaej174@gmail.com (S.E.J.); tejasree.tej17@gmail.com (T.C.J.); prasadhb@pes.edu (P.H.); gsrinivasa@pes.edu (G.S.)

**Keywords:** sparse inertial sensing, human motion reconstruction, transformers, biomechanical constraints, real-time motion capture, digital twins

## Abstract

Wearable inertial measurement units are widely used for human motion analysis due to their portability; however, most IMU-based motion capture systems rely on dense sensor configurations that increase cost, complexity, and usability challenges in real-world applications. To address this limitation, this paper presents a sparse inertial human motion reconstruction framework that uses only five wearable sensors while maintaining real-time performance and biomechanical plausibility. The proposed framework integrates Movella Xsens DOT IMUs with a learning-based inverse kinematics pipeline and a real-time biomechanical digital twin for motion reconstruction and visualization. The evaluation was conducted in two phases: first, a real-time motion streaming system was established to validate sensor alignment, coordinate frame consistency, and end-to-end latency; second, a sparse inference framework was trained using the Virginia Tech Natural Motion Dataset combined with a custom dataset containing hard negative samples. Experimental results show that the system can accurately reconstruct full-body human motion, excluding head movement, with a local Mean Per-Joint Position Error of 5.96 cm using only five sensors. Comparative ablation studies demonstrate that Transformer-based temporal modeling achieves better geometric accuracy and temporal smoothness than recurrent and convolutional baselines, while physics-informed regularization and hard negative mining significantly improve biomechanical consistency and reduce motion jitter. Real-time experiments further demonstrate that the framework operates within interactive latency limits, highlighting its potential for biomechanical digital twin applications.

## 1. Introduction

### 1.1. Background and Motivation

Human motion tracking is an essential technology used across multiple fields, including biomechanics, sports medicine, physical therapy, ergonomics, safety engineering, and human-computer interaction [1,2,3]. The increasing demand for motion sensing technologies has been driven by the need for systems that are accurate, lightweight, scalable, and capable of operating in real-world environments [4]. Applications such as patient rehabilitation monitoring and athletic performance analysis require motion capture systems that can function reliably outside controlled laboratory settings [5]. Traditional optical marker-based motion capture systems remain the gold standard for kinematic analysis [6,7]. Although these systems provide highly accurate position tracking, they depend on fixed multi-camera setups that require extensive calibration, controlled lighting conditions, and dedicated capture spaces [8]. In addition, the high financial and infrastructural costs associated with these systems limit their accessibility for small clinics, local sports centers, and educational laboratories [9]. Marker-based systems can also be intrusive, restricting natural movement and making long-term monitoring difficult [6]. These limitations highlight the need for alternative motion sensing approaches that can operate effectively beyond laboratory environments.

Wearable inertial measurement units (IMUs) have been shown to be a promising substitute for optical motion tracking systems [10]. Wearable IMUs offer the advantage of portability and do not require external infrastructure, thereby providing continuous measurements of orientation, angular velocity, and linear acceleration [11]. Wearable sensors can be used for motion tracking in various settings, such as physiotherapy clinics, sports fields, homes, and outdoor environments [12]. Nevertheless, full-body motion tracking with wearable inertial sensors may require a dense network of sensors distributed across the body, typically ranging from 10 to 17 sensors [13]. Although such systems can achieve high accuracy, they may present usability challenges, including user discomfort, longer setup times, higher costs, and reduced adherence, particularly among elderly patients or untrained users undergoing rehabilitation [14].

The key advantage of sparse IMU-based motion capture lies in its ability to eliminate the need for cameras. Whereas fusion techniques, including RGB-Depth, RGB-IMU, and IMU-LiDAR, improve performance through redundancy, they still retain a dependency on outside-in motion capture processes. Nevertheless, modern sparse IMU techniques increasingly rely on data-driven approaches, making them comparable to multimodal fusion techniques in terms of accuracy.

### 1.2. Problem Statement

Wearable motion capture technology presents a significant challenge in the accurate reconstruction of full-body human motion using only a small number of inertial sensors. A major difficulty arises from the fact that sensors may not be located on all body segments; as a result, the kinematic chain is only partially observable, making it difficult to accurately determine joint positions. Consequently, multiple poses can correspond to the same inertial data, leading to uncertainty in motion estimation. This issue is particularly important for joints such as the shoulders and hips, which play a critical role in posture, balance, and limb coordination.

In addition to limited observability, inertial sensing systems are affected by other sources of error, including sensor noise, bias drift, magnetic disturbances, and sensor placement variation [15]. These sources of error can significantly affect system accuracy, especially in sparse sensing configurations. Conventional kinematic models and filter-based approaches often fail to maintain both accuracy and stability under sparse sensing conditions unless strong assumptions are made or system responsiveness is sacrificed [16].

Such sparse sensor coverage can also result in unintended coupling between distal and proximal joints. In addition, it introduces another challenge that is often overlooked. Learning-based models may incorrectly associate high-frequency motion of distal joints (e.g., rapid wrist rotations) with the movement of proximal joints, even when the latter remain physically stationary. Addressing these challenges requires motion inference techniques that can accurately distinguish spurious correlations from actual motion.

### 1.3. Research Objectives

The principal research focus of this study is to assess whether full-body kinematics can be estimated from limited inertial measurement data. We attempt to establish a learning-based framework for reconstructing valid human motion using a minimal number of wearable inertial sensors. The study develops a sparse motion reconstruction technique formulated as a temporal learning problem that captures normal inter-joint coordination patterns from inertial sensor measurements without utilizing direct measurement techniques. The study compares various temporal modeling architectures, such as Recurrent Neural Networks (RNNs), Convolutional Neural Networks (CNNs), and Transformer-based architectures, under different sparse sensing conditions. Lastly, in order to reduce the effects of spurious distal-to-proximal joint coupling caused by variations in kinematic representations and associated learning strategies, the study demonstrates how the resulting reconstructions are validated in real time using numerical performance metrics as well as visualization of reconstructed motion on a virtual avatar.

### 1.4. Contributions

The key contributions of this work are summarized as follows:

1. We introduce a full-body motion reconstruction framework (excluding head) in real time using five IMUs on wrists, ankles, and pelvis, without using any cameras or dense sensor suits.
2. We define sparse motion reconstruction as a temporally conditioned inverse kinematics problem, which directly addresses the ill-posed nature of inferring unobserved proximal joints from distal observations alone.
3. We introduce a physics-informed Transformer network trained on a multi-objective function that penalizes angular and linear velocities without over-smoothing the motion trajectory.
4. We introduce a hard negative mining strategy using distal joint isolation tasks such as locked elbow rotation of wrist, helping the model to disentangle high-speed distal motion from erroneous proximal motion (kinematic bleed-through).

The rest of this paper is structured as follows. Section 2 discusses the related literature on sparse inertial motion capture. Section 3, Section 4 and Section 5 present the proposed framework in terms of system design, data processing, and the physics-informed Transformer. Section 6 discusses the experimental design and evaluation metrics. Section 7 presents the experimental results in terms of quantitative evaluation, ablation studies, and real-time performance benchmarking. The remaining sections, namely, Section 8, Section 9 and Section 10, discuss the implications, limitations, and future directions of the study.

## 2. Related Work

Human motion can be captured using optical, depth, and wearable inertial sensors. Each of these modalities provides unique trade-offs in terms of accuracy, usability, and deployment. These sensor modalities support a variety of motion capture methods that are extensively used in human motion analysis.

### 2.1. Dense Motion Capture Systems

Dense motion capture systems based on optical technology have been the dominant approach for capturing motion in both biomechanics and animated character production. Optical systems produce precise spatial reconstruction of 3D joint trajectories by tracking reflective markers placed on the body using multiple synchronized cameras. Optical systems are commonly used for laboratory-based biomechanical analysis, clinical gait analysis, and animated character production [6].

Although cameras enable dense motion tracking, several techniques have been specifically developed for joint identification and localization using OpenPose [17], MediaPipe [18], AlphaPose [19], and YOLO-Pose [20]. However, these algorithms are highly susceptible to external variables such as lighting conditions, image resolution, joint visibility, and distance from the camera. In addition, because these methods rely on external motion capture processes, they perform optimally in world-fixed scenarios.

Optical motion capture systems offer extremely high accuracy; however, they also present several operational challenges. These systems require a large number of cameras, each of which must be precisely calibrated. Additionally, the cameras must operate under specific lighting conditions, and the reflective markers attached to the body must remain continuously visible to the cameras. These factors can reduce tracking accuracy due to poorly controlled lighting, marker occlusion during complex movements or natural body motion, soft tissue artifacts, and limited capture areas [21].

The requirement for fixed infrastructure limits the mobility of these systems, making them unsuitable for long-term monitoring and use outside laboratory environments [22]. Researchers have explored various sensing approaches to address these limitations, with particular emphasis on wearable sensors and multimodal sensing systems. Recent research has investigated real-time human pose tracking using combinations of LiDAR and inertial measurement units (IMUs) to develop motion tracking systems that do not rely on traditional camera-based setups [23]. Other studies have explored methods for motion representation and editing, including the analysis of fine-grained joint movements and the reconstruction of realistic motion trajectories from collected sensor data [24]. These research efforts indicate growing interest in flexible motion capture frameworks capable of operating outside controlled laboratory environments.

### 2.2. Wearable Sensor-Based Motion Analysis

Motion capture and analysis using wearable inertial measurement units (IMUs) can be effectively performed without using an optical system because IMUs directly capture both linear acceleration and angular velocity. Sensor fusion algorithms are often used to estimate orientation in the form of unit quaternions, thereby avoiding singularity problems associated with Euler angles [15]. IMUs provide a means of capturing motion in uncontrolled environments such as rehabilitation clinics, sports fields, and home settings.

Early inertial motion capture systems used filtering techniques and kinematic models to estimate joint or segment orientations. Although these systems produced valid results over short periods of time, they were highly sensitive to bias drift, sensor noise, and changes in sensor position, resulting in error accumulation over time [25]. To improve performance, learning-based techniques were developed to exploit recurring temporal patterns in inertial signals. RNN and CNN models achieved improved motion estimation stability through data-driven learning and temporal modeling [26].

At the same time, the majority of existing IMU-based motion capture systems rely on dense sensor configurations typically consisting of 10–17 sensors placed on key body segments. Although these setups yield highly accurate results, their accessibility in clinical and real-world applications is limited due to increased setup complexity, higher cost, user discomfort, and calibration overhead [27].

### 2.3. Sparse Sensing Approaches in Biomechanics

The primary goal of sparse inertial motion capture is to preserve biomechanical motion while reconstructing human movement using the fewest possible wearable sensors, thereby mitigating the disadvantages of using 10–17 sensors.

In earlier sparse sensing techniques, body pose estimation was addressed by formulating constrained optimization problems that incorporated anthropometric priors and statistical body models to overcome measurement limitations. The Sparse Inertial Poser (SIP) demonstrated the feasibility of reconstructing realistic full-body poses using as few as six inertial measurement units (IMUs) by imposing kinematic and anatomical constraints [28]. However, the global optimization of full motion sequences makes these approaches computationally expensive and unsuitable for real-time applications.

Data-driven approaches have become the preferred method for posture reconstruction. Deep learning models have demonstrated that the orientations of proximal joints can be predicted using sparse inertial sensor measurements collected from both proximal and distal body segments [29]. Sequence-to-sequence architectures and recurrent neural networks are capable of achieving reasonable accuracy across a wide range of common motion scenarios [30]; therefore, they are considered viable solutions for an increasing number of real-world applications.

Recently, Transformer-based models have been explored for modeling temporal dependencies in inertial motion sequences. The Transformer Inertial Poser (TIP) utilized self-attention mechanisms to model long-range dependencies, thereby improving the temporal consistency of motion sequences over extended durations [31]. Alternative hybrid approaches incorporating additional spatial constraints or auxiliary sensors, such as ultra-wideband ranging and LiDAR, have also been used to reduce drift and improve global pose estimation [32].

Despite recent advances, several issues remain unresolved in sparse sensing systems. Persistent challenges include limited observability leading to pose ambiguity, drift accumulation, and unintended distal-to-proximal joint coupling during high-frequency isolated limb movements. With fewer sensors, these issues become increasingly difficult to resolve, highlighting the need for training strategies that explicitly account for biomechanical decoupling in sparse observations.

### 2.4. Research Gap Analysis

Existing motion capture systems have the ability to achieve high accuracy, especially because of the dense distribution of sensors and the large number of sensors working together; however, these technologies also suffer from a range of limitations regarding usability and scalability.

On the other hand, most sparse inertial methodologies capable of capturing motion using fewer inertial sensors and reduced motion capture hardware require very strong prior knowledge, computationally expensive offline optimization approaches, or highly complex hardware and software designs, thus limiting their ability to function in real-time environments. Additionally, although Transformer architectures have shown promising results for motion reconstruction, they have not yet been extensively used to address the challenges faced by sparse inertial methods. This includes understanding whether long-range temporal attention is necessary for reconstructing local kinematics. Numerical accuracy has been a primary focus of existing studies, while biomechanical plausibility, perceptual stability, and real-time digital twin validation have received comparatively less attention.

These significant gaps are driving the development of new sparse inertial motion reconstruction methods that aim to: (i) provide high reliability and usability using limited sensor configurations, (ii) reduce distal-to-proximal coupling issues encountered during human motion capture, and (iii) maintain real-time stability compatible with interactive biomechanical application environments.

## 3. System Overview

### 3.1. Overall Framework Architecture

**Figure 1:**
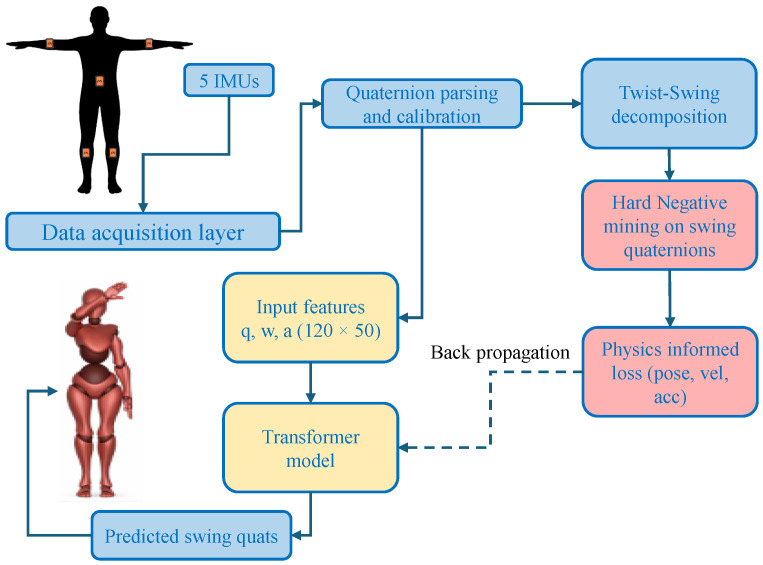
Overview of the proposed sparse inertial motion reconstruction architecture. The pipeline is organized into three primary layers: sensing, inference, and visualization.

The proposed system consists of several interconnected components that operate together to achieve real-time sparse inertial motion reconstruction. As illustrated in Figure 1, the architecture is divided into three stages. The first stage corresponds to the sensing and acquisition layer, where inertial signals are collected from a small number of IMUs attached to the body. Orientation, angular velocity, and linear acceleration measurements undergo preprocessing steps such as calibration and coordinate alignment to ensure consistency and compatibility across all sensors.

The second stage consists of the inference layer, which utilizes a temporal learning model to predict the orientations of proximal joints that are not directly measured by the sensors. The reconstructed joint rotations are subsequently applied to a humanoid skeleton and rendered in real time using forward kinematics. This modular separation into sensing, inference, and visualization layers enables the system to be easily extended to additional joints or sensing modalities without requiring modifications to the core pipeline.

The third module is the reference motion dataset consisting of hard negative samples. The relative rotations are then separated into twist and swing components. The swing rotations responsible for functional limb movement are isolated for training. During runtime, only the distal IMU measurements are fed into the temporal learning model, which predicts the proximal joint swing orientation for the next time step. The predicted orientations are normalized to ensure validity on the rotation manifold and combined with the measured sensor data to obtain the complete limb pose. The final articulated pose is displayed in real time using a Unity-based avatar, providing stable low-latency visualization. This system enables real-time motion reconstruction using minimal sensor data, while also supporting evaluation of reconstruction quality through both numerical metrics and perceptual analysis.

### 3.2. Sparse Sensor Configuration

**Figure 2:**
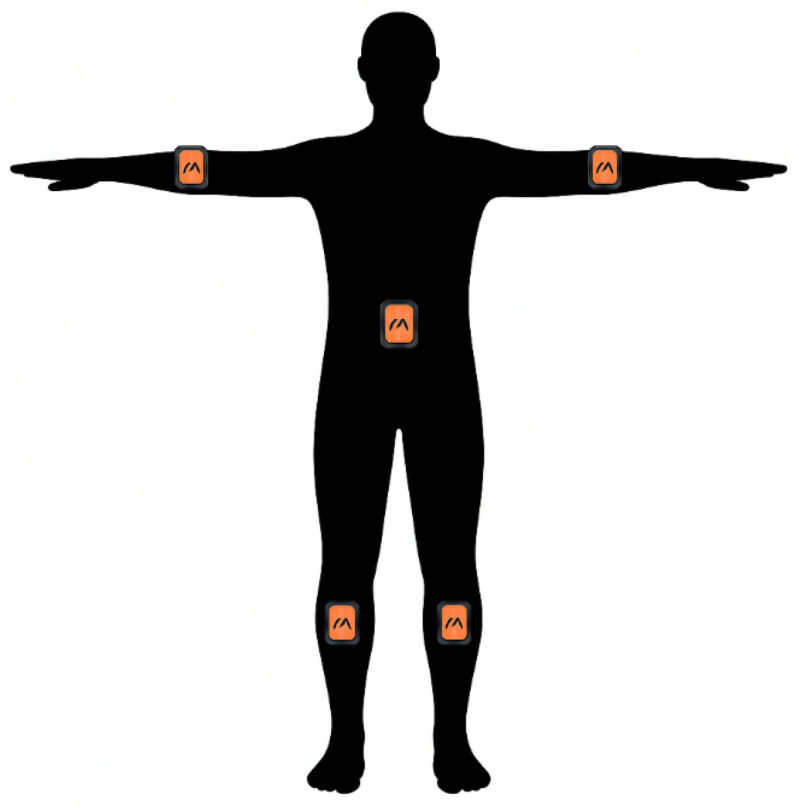
Wearable Sparse Sensing Configuration. Five inertial measurement units are deployed on the distal body segments (wrists and ankles) and the trunk (torso).

Five inertial measurement units (IMUs) are placed on the torso, both forearms, and both lower legs, as illustrated in Figure 2.

Distal limb segments provide strong kinematic cues and exhibit coordinated motion patterns that are closely related to proximal joint behavior. Within the proposed inference framework, distal limb dynamics are used as the primary source of information for motion inference, while proximal joint movements are not directly measured. Instead, proximal joint orientations are inferred by learning the relationship between distal limb dynamics and proximal joint motion. This approach is based on the biomechanical principle that distal limb segments act as kinematic integrators, accumulating the composite motion generated throughout the upstream kinematic chain. Consequently, sensor placement on distal limb segments implicitly captures latent motion information from intermediate body segments through biomechanical and kinematic constraints. The proposed configuration significantly reduces sensor requirements while maintaining biomechanical realism and a high degree of reconstruction accuracy.

## 4. Data Acquisition and Preprocessing

### 4.1. Sensor Hardware Description

We utilize the Movella Xsens DOT (2nd Gen) development kit for sparse inertial sensing. The detailed hardware specifications of the sensors are presented in Table 1, based on the manufacturer’s technical documentation [33].

### 4.2. Signal Processing and Transmission Pipeline

The stable 60 Hz output rate ensures that high-frequency motion components are preserved through orientation and velocity updates. In addition, the Movella XKFCore sensor fusion algorithm provides orientation estimates in the form of unit quaternions. The processed data is subsequently transmitted via Bluetooth Low Energy (BLE 5.0) to a central processing workstation running custom bridge software.

**Figure 3:**
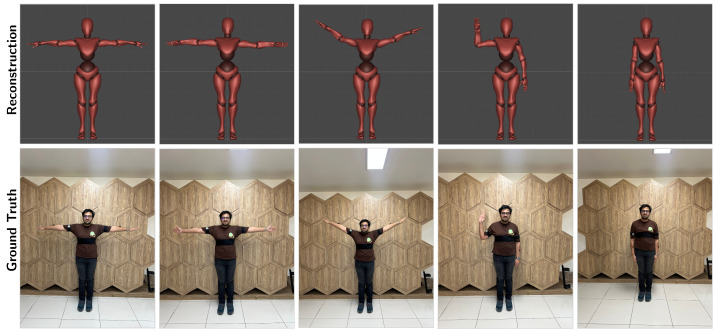
Real-Time Biomechanical Digital Twin. Visualization of the real-time motion streaming pipeline. (**Top**) The avatar is driven by sensor data in Unity. (**Bottom**) The corresponding real-world human reference.

To facilitate real-time visualization and debugging (see Figure 1 and Figure 3), we implemented a custom MQTT publisher. This middleware utilizes Movella SDK classes to manage BLE connectivity, monitor battery levels, and synchronize data packets from the five sensor nodes, thereby providing a consistent data stream for the downstream Transformer model.

### 4.3. Experimental Data Collection Protocol

During the experiments, a targeted data augmentation strategy based on hard negative mining was incorporated to mitigate Kinematic Bleed-Through (KBT). Kinematic Bleed-Through refers to a phenomenon in sparse IMU-based motion capture where the estimation of previously unobserved proximal joint configurations is influenced by artifacts propagated from distal joint movements [35]. This phenomenon creates ambiguity by allowing a single distal pose configuration to correspond to multiple valid proximal joint poses. KBT becomes particularly prominent when global root drift is ignored and only local kinematic constraints are considered [36]. The interaction between distal joint oscillations and proximal joint stability can therefore lead to erroneous motion inference, where high-frequency oscillations measured by distal sensors are incorrectly associated with proximal joint motion, thereby violating the anatomical constraints of the human kinematic chain [37].

Even though an ADL dataset contains a large number of motions that are synergistic in nature, it fundamentally lacks the degree of distal isolation required to train a versatile controller. To address this limitation, a pure motion dataset was created to include “hard negative” motions. These motions refer to statistically rare movement patterns in Activities of Daily Living (ADL) datasets that are nevertheless biomechanically valid. All data collection activities involving human participants were conducted in accordance with the Declaration of Helsinki [38]. By explicitly recording these motions, the dataset is augmented to include additional decision boundaries of human motion articulation.

To explicitly satisfy the distal-to-proximal decoupling constraint, distal joint isolation tasks were recorded. Although the dataset was not strictly limited to the following tasks, the primary recordings consisted of several highly constrained movements. First, locked-humerus elbow flexion sequences were recorded. These involved rapid sagittal forearm motions (0.5–2.0 Hz) while constraining the humerus against the ribcage. This motion specifically forces the model to decouple high-velocity forearm motions from spurious shoulder activity. Next, wrist pronation and supination motions were recorded. These involved high-frequency rotational movements with the elbow constrained to 90°. This configuration specifically minimizes wrist-to-shoulder bleed-through. Additionally, fixed-thigh knee extension tasks involving rapid shank oscillations were performed in a seated posture with a stationary femur, thereby isolating knee dynamics from hip motion.

The augmented motion dataset does not cover the entire range of human motion, but rather represents a set of specific movements. A strict tolerance criterion of |ω|<0.1 rad/s was applied to all proximal joints, ensuring that the kinematics of the recorded distal joints correctly corresponded to proximal joint stability. This constraint ensures that the Transformer model can correctly decouple high-velocity distal joint signals from false proximal joint activations. It is important to note that because the framework operates on strictly local root-relative inertial features, the kinematic representations are fundamentally motion-agnostic and decoupled from subject anthropometry. Consequently, this supplementary dataset was explicitly curated to ensure coverage of kinematic corner cases and extreme motion dynamics rather than demographic diversity.

### 4.4. Hybrid Dataset Composition

In order for the model to generalize correctly to natural human motion while also ensuring robustness to infrequent observation ambiguities, a hybrid dataset was constructed from two different sources. First, a primary training corpus was constructed from the Virginia Tech Natural Motion Dataset (VT-NMD) [27], which also serves as the standard ground truth for natural human kinematics in this study. The base dataset consists of approximately 40 h of unscripted, open-world Activities of Daily Living (ADLs) captured using an industry-standard Xsens MVN Link full-body inertial motion capture suit consisting of 17 sensors. The dataset encompasses a broad range of unscripted, open-world ADLs, capturing diverse motion types including locomotion, object interaction, physical exercise, and household activities [27].

Using the provided processing code for data analysis [27], the full-body kinematic chain was extracted from the source data, providing ground truth orientations for the proximal joints, including the upper arms and thighs. A “sparse mask” was applied to the dataset, retaining only the quaternion and inertial measurements corresponding to the five target sensor locations (see Figure 2).

Furthermore, the natural motion dataset was augmented with the “hard negative” injection dataset recorded according to the protocol described in Section 4.3. This dataset specifically targets high-frequency distal joint isolation movements and serves as targeted correction data during training. During training, a hard negative injection ratio of approximately ≈10% was used, where correction data was injected into every training batch (Section 7.3.3). Lower injection ratios empirically resulted in false positives in the form of persistent proximal activation during rapid distal motion, whereas higher injection ratios negatively affected performance on general Activities of Daily Living.

### 4.5. Computational Preprocessing and Feature Engineering

As a result of the above steps, the raw sensor streams are then subjected to a rigorous computational preprocessing pipeline, which ensures spatial consistency, numerical stability, and kinematic isolation prior to network inference. This pipeline can be broadly categorized as frame calibration, signal sanitization, and geometric feature engineering.

#### 4.5.1. Calibration & Frame Transformation

Prior to every data session, a static T-pose calibration was conducted to align the sensor coordinate frames with the corresponding anatomical body frames. This alignment ensures that the input quaternion $q_{\mathrm{input}}$ represents the limb orientation relative to a gravity-aligned global reference frame, invariant to arbitrary sensor mounting placement on the skin. The frame transformation pipeline implemented by the controller middleware consists of two mathematical operations.

First, a coordinate system permutation denoted by $T_{\mathrm{coord}}$ is applied. The raw IMU quaternions, $q_{\mathrm{raw}}=\left[w,x,y,z\right]$, are represented in the sensor’s right-handed East-North-Up (ENU) coordinate frame. To convert this into the global coordinate frame of the application (Unity, left-handed, Y-up), a basis permutation is applied. In accordance with limb-specific mounting conventions, the permutation function $T_{\mathrm{coord}}\left(q_{\mathrm{raw}}\right)$ yields the global orientation $q_{\mathrm{global}}$ as

(1) $$q_{\mathrm{global}}=\left\{\begin{array}{cc} \left[w,y,-x,z\right] & \mathrm{for}\;\mathrm{upper}\;\mathrm{body}\;\mathrm{segments}\\ \left[w,y,-x,-z\right] & \mathrm{for}\;\mathrm{lower}\;\mathrm{body}\;\mathrm{segments} \end{array}\right.$$

The sign difference in the z-axis component is meant to account for the sensor mounting orientation relative to the limb’s proximal joint axis, ensuring consistent alignment with the virtual world’s vertical axis.

Subsequently, the boresight alignment step ($T_{\mathrm{calib}}$) is carried out to account for the sensor mounting variability, essentially capturing the unique angular offset of how each sensor is strapped to the user. We define the orientation established during the T-pose at time $t_{0}$ as $q_{\mathrm{global}}\left(t_{0}\right)$. The static boresight offset, $q_{offset}$, is computed by taking the inverse of this orientation:

(2) $$q_{\mathrm{offset}}=q_{\mathrm{global}}{\left(t_{0}\right)}^{-1}=\left[w_{0},-x_{0},-y_{0},-z_{0}\right]$$

During dynamic motion at any time (t>0), the true calibrated orientation $q_{calibrated}\left(t\right)$ is computed by the following Hamilton product (⊗):

(3) $$q_{\mathrm{calibrated}}\left(t\right)=q_{\mathrm{offset}}⊗q_{\mathrm{global}}\left(t\right)$$

This sequence ensures that $q_{\mathrm{calibrated}}\left(t_{0}\right)=\left[1,0,0,0\right]$ at the calibration instant, effectively removing the sensor mounting bias and measuring the true anatomical rotation of the limb segment relative to the reference pose.

#### 4.5.2. Signal Preprocessing and Temporal Segmentation

Raw sensor data is subjected to a rigorous cleaning and formatting pipeline, which ensures numerical stability for the Transformer architecture. First, the internal 800 Hz sensor data is downscaled using Strap-Down Integration (SDI) to 60 Hz [33], thereby matching the rendering frame rate of the Unity client. Second, all data streams are subjected to a scan for numerical anomalies. In this regard, files containing infinite values or extensive NaN sequences are automatically detected and removed from the training pipeline in order to avoid gradient explosion. Thirdly, the continuous time-series data is segmented into fixed-length sequences using a sliding window approach to capture temporal features. A sequence length (*L*) of 120 frames, or 2.0 s, is applied, which is sufficient to capture multi-joint coordination while maintaining compatibility with real-time inference constraints.

#### 4.5.3. Feature Representation and Target Reparameterization

A specific set of geometric and inertial features is extracted, chosen with the goal of decoupling the proximal and distal kinematic chains. For each of the five instrumented segments, a 10-dimensional feature vector ($x_{t}^{\left(i\right)}\in R^{10}$) is extracted at each time step *t*. This local feature vector consists of three parts: the unit quaternion ($q\in R^{4}$), representing the orientation of the limb with respect to the global frame; the calibrated angular velocity ($\omega \in R^{3}$); and the calibrated linear acceleration ($a\in R^{3}$).

To ensure numerical stability and maintain proper gradients during the training process, different normalization techniques are applied to different feature groups. The unit quaternions are strictly L2-normalized, thereby maintaining the unit norm constraint on the SO(3) manifold. In contrast, global z-normalization is applied to the inertial features $\omega_{\mathrm{distal}}$ and $a_{\mathrm{distal}}$. This choice of standardization technique is motivated by the high-kurtosis nature associated with rapid impact accelerations.

In this context, conventional min-max normalization was found to be insufficient. To address this limitation, a standardized normalization strategy with outlier clamping was employed to map the features into a numerically stable range suitable for Transformer training:

(4) $$x_{norm}=\mathrm{clamp}\left(\frac{x-\mu }{\sigma +\epsilon },-5.0,5.0\right)$$

where x represents the raw input feature, while μ∈R and σ∈R denote the scalar global mean and standard deviation, respectively. To preserve the intrinsic 3D structure of inertial vectors, these statistics are computed uniformly across all three spatial axes (x,y,z) for each sensor modality (e.g., a shared μ and σ for the complete 3D acceleration vector associated with a given sensor), rather than independently for each axis. Because identical normalization statistics are applied uniformly across all spatial components of a given sensor modality, relative directional consistency between the three axes is preserved while the signal scale is normalized. The clamping threshold of ±5σ was selected to account for rare yet biomechanically valid high-velocity motions used as hard negative samples to train the network. The threshold covers approximately 99.9999% of the distribution, ensuring that extreme yet physically plausible kinematics are retained while removing non-biological outliers that may result from sensor impacts or hardware transients.

**Figure 4:**
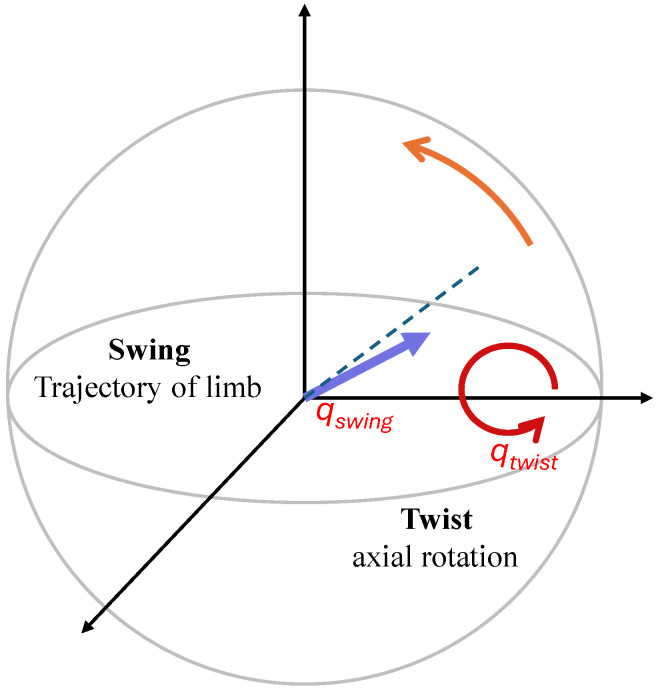
Geometric swing-twist decomposition of relative limb orientation ($q_{rel}$). Decomposing rotations into swing ($q_{swing}$) and twist ($q_{twist}$) components effectively disentangles the limb’s spatial trajectory from noisy axial sensor data.

Subsequent to this step, the prediction target is reparameterized via a twist-swing decomposition (Figure 4) to decouple anatomically meaningful motion components. The orientations of the proximal and distal limbs are described using unit quaternions $q_{proximal}$ and $q_{distal}$, respectively.

The process starts with computing the relative rotation needed to align the distal frame to the proximal frame to isolate joint articulation from global heading:

(5) $$q_{rel}=q_{proximal}⊗q_{distal}^{-1}$$

where ⊗ denotes the Hamilton quaternion product and $q_{distal}^{-1}$ denotes the quaternion inverse (conjugate) of the distal quaternion.

Next, the twist component is extracted according to the twist-swing decomposition principles described in Motion-Sphere [24]. We define the anatomical twist axis of the limb, u, as the x-axis vector $u={\left[1,0,0\right]}^{T}$. Given that *w* is the scalar part and $v={\left[x,y,z\right]}^{T}$ is the vector part of the relative rotation, we extract the twist component (rotation around the limb axis) by projecting v onto u:

(6) $$p_{proj}=\left(v\cdot u\right)u$$

The non-normalized twist quaternion is constructed as $q_{raw\_twist}=\left[w,p_{proj}\right]$. The final unit twist quaternion is

(7) $$q_{twist}=\frac{q_{raw\_twist}}{∥q_{raw\_twist}∥}$$

Finally, the swing component, representing the trajectory of the limb axis (the pointing direction) independent of axial rotation, is isolated according to the formulations in Motion-Sphere [24]. Since the total rotation is the composition of swing and twist ($q_{rel}=q_{swing}⊗q_{twist}$), we isolate the swing component $q_{swing}$ by multiplying by the inverse twist:

(8) $$q_{swing}=q_{rel}⊗q_{twist}^{-1}$$

The model is trained to minimise the error between the predicted swing ${\hat{q}}_{swing}$ and its ground truth $q_{swing}$. This helps to mitigate noise in axial rotation, which is typically seen in wrist-worn sensors during sparse observation tasks.

## 5. Proposed Sparse Sensing Methodology

### 5.1. Sparse Observability Problem

The main challenge of this research is to solve a learning-based inverse kinematics reconstruction problem under conditions of extreme observational sparsity. We define the complete internal kinematic state of the human body at time *t* as $S_{t}=\left\{J_{\mathrm{proximal}}^{t},J_{\mathrm{distal}}^{t}\right\}$, where each joint state J contains both orientation and inertial information. The observable input $X_{t}$ is defined as a feature embedding derived from $J_{\mathrm{distal}}^{t}$ and the torso reference.

In a standard Motion Capture (MoCap) setup (e.g., Xsens MVN Link), the observation function $O_{\mathrm{full}}$ is bijective and represents the complete state space:

(9) $$Z_{t}=O_{\mathrm{full}}\left(S_{t}\right)\approx S_{t}$$

where $Z_{t}$ denotes the observed sensor measurements and $S_{t}$ represents the underlying kinematic state at time *t*.

**Figure 5:**
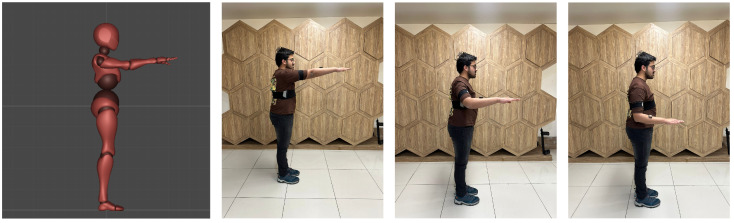
Visualization of the Sparse Observability Problem. A single distal observation (wrist orientation) corresponds to multiple valid proximal configurations.

However, in the proposed framework, the observation function $O_{\mathrm{sparse}}$ plays the role of a masking operator, allowing only the distal states (end effectors) and the root reference (torso) to be visible, while making the proximal states latent. The absence of secondary sensors on the proximal limb segments and global position information (e.g., UWB anchors and optical cameras) leads to a fundamental ill-posed problem. We formally define this condition as *Sparse Observability*. Under this condition, a single instantaneous distal observation (e.g., wrist orientation) is associated with a continuous manifold of valid proximal configurations (e.g., varying elbow swivel angles). As illustrated in Figure 5, this motion duality renders the inverse kinematic problem unsolvable at any single time step.

To resolve this, we formalize the reconstruction task as learning a mapping function $F_{\theta }$ (parameterized by the Transformer) that estimates the conditional probability of the proximal state, given a temporal history of distal observations:

(10) $$P(J_{\mathrm{proximal}}^{t}|X_{t-L:t})$$

In practice, this conditional distribution is approximated via deterministic regression using an encoder-only Transformer. The network is trained to minimize the expected reconstruction error over the proximal joint space by mapping the input tensor sequence $X_{t-L:t}$ (a temporal context of length *L* containing features from the five observed IMUs) directly to the target state vector $J_{\mathrm{proximal}}^{t}$ for the uninstrumented proximal joints (e.g., shoulders and thighs) at time *t*.

### 5.2. Temporal Modeling of Motion Dynamics

In addressing the physics observability and motion duality challenges, wherein an instantaneous distal joint configuration has multiple corresponding valid configurations of the proximal joint, we formulate the reconstruction problem as a Sequence-to-Vector regression task. In this way, the system can leverage the temporal history of dynamics in distal joint configurations to resolve the ambiguity in proximal joint configurations.

#### 5.2.1. Input Phase Space Formulation

The input sequence $X\in R^{L\times D_{in}}$ is defined as a sliding window containing the current frame *t* and the preceding L−1 frames (i.e., the interval [t−L+1,…,t]) of the sequence history. Because this buffer contains no future observations, it guarantees zero algorithmic look-ahead delay, ensuring true real-time causality. Through an extensive empirical ablation study (as discussed in Section 7.3.4), an optimal context window length L=120 frames, or 2.0 s at 60 Hz, was chosen. This time window is critical in addressing the multi-scale aspects of human biomechanics, as it must be long enough to capture the low-frequency preparation phases of the movement (e.g, the subtle torso lean preceding a reach) yet also short enough to capture the high-frequency inertial content of the movement necessary to detect rapid oscillations in the distal joint.

The dimension of the input sequence $D_{in}$ is set to 50. The feature dimension at each time step *t* is formed by concatenating the 10-dimensional kinematic states of all five instrumented segments. For each sensor i∈{1…5}, the individual feature vector is defined as

(11) $$x_{t}^{\left(i\right)}={\left[q_{t}^{\left(i\right)},\omega_{t}^{\left(i\right)},a_{t}^{\left(i\right)}\right]}^{T}$$

where $q_{t}^{\left(i\right)}\in R^{4}$ is the unit quaternion describing the orientation of the sensor with respect to the global vertical frame and $\omega_{t}^{\left(i\right)}\in R^{3}$ and $a_{t}^{\left(i\right)}\in R^{3}$ are the normalized angular velocity and linear acceleration, respectively. These are concatenated to form the 50-dimensional input, $X_{t}=\mathrm{Concat}\left(x_{t}^{\left(1\right)},\ldots ,x_{t}^{\left(5\right)}\right)$. Consequently, the final input tensor provided to the Transformer is of the shape (*B*, 120, 50), where *B* represents the batch size.

Crucially, these inertial derivatives are not merely auxiliary; they provide the high-frequency jitter signals that the Transformer’s attention mechanism utilizes to explicitly identify the relationship between distal joint chaos and proximal joint stability.

#### 5.2.2. Temporal Injection via Positional Encoding

The permutation-invariance property of the basic Transformer architecture necessitates the injection of explicit temporal ordering into the input embeddings. Standard sinusoidal Positional Encodings (PE) [39] are directly added to the input embeddings to preserve the sequential nature of the motion. For a position pos and dimension 2i or 2i+1,

(12) $$\begin{array}{cc} PE_{\left(pos,2i\right)} & =\sin\left(\frac{pos}{10000^{2i/d_{model}}}\right) \end{array}$$

(13) $$\begin{array}{cc} PE_{\left(pos,2i+1\right)} & =\cos\left(\frac{pos}{10000^{2i/d_{model}}}\right) \end{array}$$

In these equations, $d_{model}$ represents the constant latent dimensionality of the model (set to 256). The continuous nature of this PE allows the model to attend to differences in temporal context. This allows the Transformer to distinguish between immediate dynamic events, such as a sudden impact jerk at time *t*, and the causal history of the movement, such as the velocity initialization that occurred at time t−50.

### 5.3. Learning Architecture

**Figure 6:**
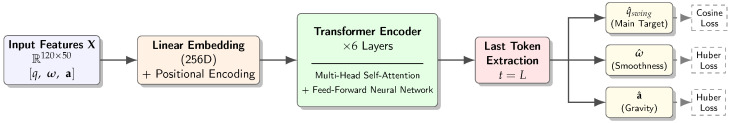
Schematic of the attention-guided encoder-only Transformer-based architecture for biomechanically constrained sparse motion reconstruction.

A standard encoder-only variant of the Transformer architecture shown in Figure 6 is employed for high-fidelity signal reconstruction. The selection of the Transformer architecture over established sequential models (such as LSTMs or GRUs) is motivated by a structural requirement of the Kinematic Bleed-Through (KBT) problem. The Transformer’s Multi-Head Self-Attention (MSA) mechanism provides a constant maximum path length between any two frames within the 120-frame temporal window [39]. This global receptive field avoids recursive state propagation, allowing the model to jointly process long-range dependencies, such as low-frequency preparatory motions and high-frequency distal transients across the full observation horizon. This structural property is particularly well-suited for leveraging the hard negative training distribution, enabling the network to associate distal instability with proximal kinematic stability.

#### 5.3.1. Network Topology

As depicted in Figure 6, the architectural backbone consists of N=6 identical layers of the Transformer encoder with a latent dimension $d_{model}=256$ and h=8 attention heads.

This architecture results in a total of 4,743,946 trainable parameters (≈4.74 M), being sufficient to represent the complex kinematic dependencies between the inputs while keeping the architecture computationally efficient enough to be used for real-time processing at 60 Hz. The temporal modeling module is based on the original Transformer encoder architecture proposed by Vaswani et al. [39], which also underlies the positional encoding formulation described in Section 5.2.2. This design choice was made deliberately over more recent efficient Transformer variants (e.g., sparse-attention or sequence-reduction models such as Longformer or Informer) for two key reasons.

First, such architectures are primarily designed to alleviate the $O(N^{2})$ computational complexity associated with very long input sequences. In our setting, the temporal context is strictly limited to L=120 frames (≈2.0 s), for which the quadratic attention cost is negligible.

Second, the proposed system is explicitly constrained by real-time latency requirements (<10 ms). Introducing additional mechanisms such as sparse attention routing or hierarchical pooling would increase algorithmic complexity without providing measurable benefits at this sequence scale. Consequently, a lightweight vanilla self-attention formulation was selected to maintain computational efficiency while preserving modeling fidelity.

Each layer of the encoder uses a Pre-LayerNorm configuration to improve the training stability of deeper layers. Each encoder layer consists of two components: (i) Multi-Head Self-Attention (MSA) followed by (ii) a position-wise Feed-Forward Network (FFN).

The Multi-Head Self-Attention (MSA) module aggregates value vectors through attention weights computed from query–key interactions. Mathematically, for a single attention head, the attention operation can be written as

(14) $$\mathrm{Attention}\left(Q,K,V\right)=\mathrm{softmax}\left(\frac{QK^{T}}{\sqrt{d_{k}}}\right)V$$

where Q, K, and V represent the Query, Key, and Value matrices derived from the input motion features and $d_{k}$ represents the dimensionality of each attention head. This operation yields a context-weighted representation of the sequence, capturing the relevance of past frames to the current pose. For example, the model may refer to a torso lean in the past to correctly predict a shoulder reach in the present.

Rather than utilizing explicit frequency-filtering modules, the mitigation of Kinematic Bleed-Through (KBT) is a data-driven emergent behavior of the standard Multi-Head Self-Attention mechanism. By training on the hybrid dataset containing targeted distal-isolation tasks, the network inherently learns to associate high-frequency oscillatory features of the distal input ($\omega_{distal}$, e.g., rapid wrist shaking) and predicts the stationarity of the proximal input. Effectively, chaos in the lower arm is attended to as a signal for stability in the upper arm, allowing the model to gate the propagation of distal noise and prevent temporally inconsistent high-frequency inputs from corrupting proximal joint predictions.

The Position-wise Feed-Forward Network (FFN) applies a fully connected nonlinear transformation to each position independently, with an expansion dimension of $d_{ff}=1024$ and GELU activation. Here, $W_{1}$ and $W_{2}$ are the trainable weight matrices, while $b_{1}$ and $b_{2}$ are the corresponding bias vectors:

(15) $$\mathrm{FFN}\left(x\right)=\mathrm{GELU}\left(xW_{1}+b_{1}\right)W_{2}+b_{2}$$

#### 5.3.2. Multitask Projection Head

To maintain strict temporal causality without requiring autoregressive triangular masking, the architecture operates as a Sequence-to-Vector model. The final latent representation of the last time step ($z_{L}$, corresponding to time *t*) is isolated and projected via parallel linear heads to reconstruct the full kinematic state of the four uninstrumented proximal joints (i.e., the left and right upper arms and left and right upper legs), treating the reconstruction as a Multi-Task Learning (MTL) problem. Specifically, for each joint, the network predicts the swing quaternion (${\hat{q}}_{swing}\in R^{4}$) as the primary orientation target decoupled from axial twist, the angular velocity ($\hat{\omega }\in R^{3}$) to enforce temporal smoothness constraints, and the linear acceleration ($\hat{a}\in R^{3}$) to ensure consistency with gravity vectors. Because the model predicts these 10 features for each of the 4 joints, the final output tensor shape of the multi-task projection head is (B×40), where *B* represents the batch size.

#### 5.3.3. Physics-Informed Multi-Task Learning (MTL) Objective

To enforce biomechanical plausibility and model a kinematically generalizable, motion-agnostic controller capable of handling both ADLs and high-frequency distal joint isolation tasks, we minimize a composite MTL loss function:

(16) $$L_{MTL}=\lambda_{q}L_{quat}+\lambda_{\omega }L_{vel}+\lambda_{a}L_{acc}$$

This objective consists of three distinct components.

Manifold-Aware Orientation Loss ($L_{quat}$) utilizes a cosine similarity loss formulation:

(17) $$L_{quat}=1-\left|\hat{q}\cdot q_{gt}\right|\;\left(\lambda_{q}=1.0\right)$$

where $\hat{q}$ denotes the predicted unit quaternion and $q_{gt}$ represents the ground-truth orientation. This formulation accounts for the antipodal symmetry (double-cover property) of the unit quaternion hypersphere $S^{3}$, where *q* and −q represent identical rotations in SO(3). Unlike Euclidean metrics (e.g., MSE), it ensures invariance to global sign ambiguity (q≡−q).

Stationarity soft constraint ($L_{vel}$) acts as the driver for our hard negative mining strategy. A penalty ($\lambda_{\omega }=0.1$) is applied to angular velocity errors during hard negative batches (selected via hyperparameter ablation in Section 7.3.2). To reduce sensitivity to high-kurtosis inertial spikes, the loss is formulated using Smooth L1 (Huber) regularization rather than standard MSE. For predicted 3D angular velocity $\hat{\omega }$ and ground truth $\omega_{gt}$ across all *J* proximal joints and Cartesian components d∈{x,y,z},

(18) $$L_{vel}=\frac{1}{3J}\sum_{j=1}^{J}\sum_{d\in \{x,y,z\}}\left\{\begin{array}{cc} 0.5{\left({\hat{\omega }}_{j,d}-\omega_{gt,j,d}\right)}^{2} & \mathrm{if}\;|{\hat{\omega }}_{j,d}-\omega_{gt,j,d}|\;<\;1\\ |{\hat{\omega }}_{j,d}-\omega_{gt,j,d}|\;-\;0.5 & \mathrm{otherwise} \end{array}\right.$$

This Huber loss formulation ensures that small errors are treated quadratically for precision, while large inertial spikes, commonly observed in rapid wrist movements, are penalized linearly to reduce sensitivity to non-biological outliers.

In sparse inertial setups lacking global position references (e.g., cameras or UWB), enforcing hard zero-velocity constraints can induce integral drift accumulation. KBT is therefore mitigated as a *physics-guided soft constraint*, encouraging drift suppression while preserving the micro-adjustments required for kinematic continuity without additional proximal joint instrumentation.

Auxiliary regularization ($L_{acc}$) introduces a minor loss term ($\lambda_{a}=0.01$) (Section 7.3.2) to encourage trajectory smoothness and gravity-vector consistency in linear acceleration predictions. The loss is similarly computed using Smooth L1 (Huber) regularization:

(19) $$L_{acc}=\frac{1}{3J}\sum_{j=1}^{J}\sum_{d\in \{x,y,z\}}\left\{\begin{array}{cc} 0.5{\left({\hat{a}}_{j,d}-a_{gt,j,d}\right)}^{2} & \mathrm{if}\;|{\hat{a}}_{j,d}-a_{gt,j,d}|\;<\;1\\ |{\hat{a}}_{j,d}-a_{gt,j,d}|\;-\;0.5 & \mathrm{otherwise} \end{array}\right.$$

where $\hat{a}$ and $a_{gt}$ denote the predicted and ground-truth 3D linear accelerations, respectively.

### 5.4. Post-Processing & Kinematic Constraints

While the Transformer effectively models the temporal dynamics of human motion, raw neural network predictions can occasionally violate physiological limits, particularly in unseen scenarios. To further enforce biomechanical validity and constrain the motion duality manifold (Section 5.1), we implement a lightweight post-processing stack at inference.

First, conservative anatomical constraints are applied. Since the model predicts relative orientations, we enforce kinematic constraints by mapping the relative quaternion into a local joint-aligned Euler parameterization, apply bounded constraints, and re-project the result back onto SO(3). This step acts as a deterministic complement to the data-driven emergent behavior of the attention mechanism (Section 5.3). While the Transformer learns to minimize Kinematic Bleed-Through (KBT) via soft constraints, this hard clamping layer ensures that residual bleed-through does not result in biomechanically invalid poses. Specifically, the elbow is modeled as a hinge joint with relative flexion $\theta_{elbow}$ constrained to [5°, 150°] to prevent hyperextension or physically impossible flexion. The glenohumeral (shoulder) joint is restricted within an angular bounding box (e.g., Pitch/Yaw/Roll axes bounded within [−45°, 150°]) to prevent the arm from clipping through the torso. To mitigate the risk of gimbal lock, a known limitation of Euler parameterizations, the local joint coordinate frames are strictly pre-aligned during the static calibration phase (Section 4.5.1) such that the intermediate rotation-axis singularity (e.g., pitch → ±90°) is shifted away from the dominant anatomically reachable workspace of the shoulder. Because Euler parameterization is used only transiently for constraint enforcement and the filtered state remains represented in quaternion space, the continuous motion trajectory is not propagated through Euler singularities.

These conservative limits are chosen to ensure physiological plausibility without over-restricting the natural range of motion.

Following this, temporal smoothing is performed using a simplified lightweight scalar-gain implementation of the Multiplicative Extended Kalman Filter (MEKF) to eliminate high-frequency jitter commonly observed during frame-by-frame regression. In this formulation, a scalar gain $K_{k}$ is used for each frame *k* under the assumption that rotational uncertainty is approximately isotropic across the three principal axes of each joint. The filter dynamically blends the quaternion component of the Transformer’s raw prediction (treated as the quaternion observation $q_{k}^{obs}\in S^{3}$ for each joint), with the prior estimate (${\hat{q}}_{k-1}$) using a constant process noise ($\sigma_{p}^{2}=10^{-6}$) and measurement noise variance ($\sigma_{m}^{2}=10^{-3}$) to compute the scalar Kalman gain $K_{k}$:

(20) $$K_{k}=\frac{P_{k-1}+\sigma_{p}^{2}}{P_{k-1}+\sigma_{p}^{2}+\sigma_{m}^{2}}$$

(21) $${\hat{q}}_{k}=\exp\left(K_{k}\log\left(q_{k}^{obs}⊗{\hat{q}}_{k-1}^{-1}\right)\right)⊗{\hat{q}}_{k-1}$$

Here, $P_{k-1}$ is the prior error covariance, ⊗ denotes the Hamilton product, and the log and exp operators map the rotational residual between SO(3) and its tangent space. This multiplicative update preserves rotational geometry while maintaining unit norm up to numerical precision.

This acts as an adaptive low-pass filter, smoothing motion while maintaining lower latency than standard moving-average techniques.

### 5.5. Real-Time Inference & Calibration Mechanism

The system architecture decouples the deep learning inference engine from the Unity-based visualization client through an asynchronous MQTT bridge, enabling modularity and fault tolerance.

**Figure 7:**
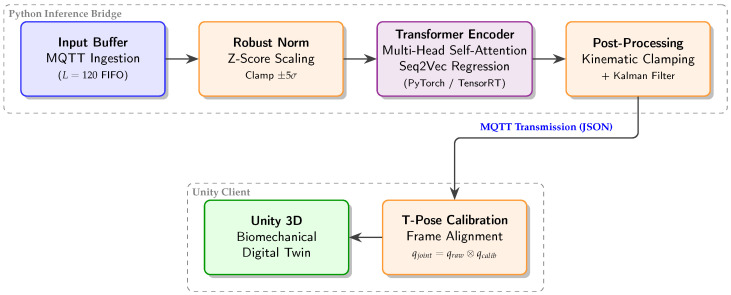
Pipeline for real-time motion inference and visualization. Our system separates the real-time inference engine from the Unity client using an asynchronous MQTT bridge. Sensor data is processed through buffering, normalization, and Transformer-based regression, followed by post-processing and T-pose calibration for visualization. This modular approach provides a stable and low-latency motion reconstruction suitable for real-time interaction (60 Hz).

The real-time inference loop is managed by an inference bridge that maintains a sliding window buffer of length L=120 (Figure 7), where distal sensor packets are ingested via MQTT and inserted into a first-in-first-out (FIFO) queue for processing. Once the buffer has enough data, normalization statistics, as described in Section 4.5.3, are calculated immediately and then applied to the incoming data before it is sent to the Transformer for inference.

While initial stress-testing of the pipeline was performed on an NVIDIA RTX 4090, the lightweight nature of the 4.74M-parameter model allows efficient execution on standard hardware. Benchmarking on a consumer-grade CPU (Intel Core i7-12700H) yields an average forward-pass inference latency of approximately 4.2 ms. This comfortably satisfies the 16.6 ms latency budget required for 60 Hz real-time rendering, demonstrating that the proposed framework remains practical without requiring high-end GPU acceleration.

In the absence of global position tracking, the unified skeleton reference is established through a relative alignment mechanism, which maps the coordinate frames of the sensors to the avatar skeletal structure. This process is done by the following steps: the user must perform the T-Pose calibration, standing with their arms extended and palms facing downwards. During this process, the instantaneous orientations of the physical sensor $q_{sensor}^{Tpose}$ and the corresponding avatar joint $q_{avatar}^{Tpose}$ are recorded. The calibration offset for each sensor is calculated using the following equation:

(22) $$q_{calib}={\left(q_{sensor}^{Tpose}\right)}^{-1}⊗q_{avatar}^{Tpose}$$

At runtime, this offset is applied to the raw sensor orientations $q_{raw}$ to map them onto the avatar frame:

(23) $$q_{joint}=q_{raw}⊗q_{calib}$$

In practice, this keeps the reconstructed pose aligned with the user, even when there are small inconsistencies in how the sensors are mounted.

## 6. Experimental Setup

### 6.1. Experimental Design

The experimental evaluation is designed to rigorously assess the fidelity of estimating unobserved proximal joint configurations using only sparse distal observations.

A key distinction of this work lies in the definition of the evaluation space. Since the proposed system is not augmented with external global position tracking systems, such as optical cameras, LiDAR, or ultra-wideband (UWB) sensors, all reconstruction metrics are defined strictly in a local root-relative frame. Specifically, all joint positions and orientations are reconstructed within a Body-Centric Reference Frame (BCRF) relative to the sternum sensor. This formulation explicitly separates global translation from articulated body motion, enabling evaluation of biomechanical pose reconstruction fidelity, including motions such as elbow flexion and shoulder abduction, without being affected by the global accumulated drift inherent to inertial dead reckoning. Consequently, the reported evaluation metrics consist of local position-based measures, including Mean Per Joint Position Error (MPJPE) and Percentage of Correct Keypoints (PCK).

To mitigate the observability problem described in Section 5.1, where a single distal pose may correspond to multiple proximal joint configurations, the experimental design incorporates hypothesis space reduction through temporal aggregation. By utilizing a temporal context window of L=120 frames (approximately 2.0 s), the manifold of feasible motion solutions is constrained, allowing evaluation of whether the selected temporal horizon is sufficient to resolve the motion ambiguity inherent in sparse sensing.

Furthermore, the models were trained and evaluated using the hybrid dataset constructed from VT-NMD Activities of Daily Living (ADLs) and the supplementary hard negative dataset. Unless otherwise specified in the subsequent ablation studies, all reported results utilize a standard configuration consisting of a 10% hard negative injection ratio and a temporal context length of L=120. This configuration was empirically determined to provide the optimal trade-off between smooth ADL motion reconstruction and high-frequency transient motion capture, as discussed further in Section 7.3.

### 6.2. Baseline Methods: Analysis of Temporal Backbone

Since most state-of-the-art sparse reconstruction methods report global pose errors that inherently include root drift, a direct numerical comparison with external baselines is scientifically inappropriate under the strict local evaluation protocol adopted in this work (Section 6.1). Therefore, to isolate the effectiveness of the proposed Transformer architecture, a controlled analysis of the temporal backbone was performed within the proposed framework.

All evaluated models were trained under an identical configuration comprising a sliding window of L=120 frames (120×50 features), the hybrid dataset with a 10% hard negative injection ratio, and the physics-informed multi-task loss function ($L_{MTL}$, detailed in Section 5.3.3). This setup ensures that performance variations can be attributed directly to the ability of each architecture to model complex temporal dependencies and resolve the motion ambiguity problem.

To evaluate these temporal dependencies, a diverse set of sequential architectures was implemented and tested. First, a simple Recurrent Neural Network (RNN) was evaluated as a baseline for basic temporal recurrence, specifically to determine whether short-horizon dependencies require only hidden-state propagation or more advanced gating mechanisms. Subsequently, two-layer variants of Long Short-Term Memory (LSTM) and Gated Recurrent Unit (GRU) architectures were employed. The LSTM model served as the primary baseline for evaluating the effect of gating mechanisms (input, forget, and output gates) on gradient propagation across the full temporal horizon of the L=120 window. In contrast, the computationally efficient GRU architecture was evaluated to determine whether simplified gating mechanisms could adequately capture the required biomechanical constraints using fewer parameters. Furthermore, a two-layer Bidirectional LSTM (BiLSTM) was employed to assess the contribution of non-causal future context within the sliding window. By processing the sequence in both forward and reverse directions, the BiLSTM potentially captures additional temporal dependencies that contribute to smoother reconstructed motion trajectories.

In addition to the recurrent models, a Temporal Convolutional Network (TCN) was implemented as a convolutional baseline using dilated causal convolutions to capture local and mid-range temporal dependencies. The model employed a kernel size of 3 together with exponentially increasing dilation factors (d=1,2,4,…) to emulate a wide temporal receptive field. This architecture was designed to evaluate whether hierarchical local feature extraction is sufficient for motion reconstruction without requiring the global pairwise attention mechanism used in Transformer architectures.

Finally, these baselines were compared against the proposed encoder-only Transformer architecture (Section 5.3). Unlike the recurrent and convolutional variants, the Transformer utilizes Multi-Head Self-Attention to dynamically weight the importance of each time step across the entire temporal window. It is hypothesized that this global receptive field is critical for identifying and suppressing the high-frequency distal joint oscillations associated with kinematic bleed-through.

### 6.3. Evaluation Metrics

For thorough evaluation of the geometric, spatial, and temporal accuracy of the reconstructed motion, we use a comprehensive set of evaluation metrics. All evaluation metrics are computed in the Local Body-Centric Reference Frame (BCRF) to eliminate global drift and focus on articulation quality only. Unless otherwise stated, all reported scalar metrics correspond to the global mean over the entire test set, averaged across all *T* time steps and all *J* predicted joints. To further isolate reconstruction error from inter-subject anthropometric variation, skeleton retargeting is applied to normalize limb lengths between the ground-truth subject and the prediction model.

In the context of training objective, standard Euclidean losses (e.g., MSE) are ill-defined on the unit quaternion hypersphere $S^{3}$ due to the double-cover property, where q and −q represent the same physical rotation in SO(3). To respect the geometry of the rotation manifold, we adopt an inner-product (cosine) quaternion loss, which is invariant to antipodal symmetry and smooth on $S^{3}$, as commonly used in the motion modeling literature [40]. Let ${\hat{q}}_{t}\in S^{3}$ denote the predicted unit quaternion and $q_{t}\in S^{3}$ the ground-truth orientation at time *t*. The manifold-aware loss is defined as

(24) $$L_{\mathrm{quat}}=\frac{1}{T}\sum_{t=1}^{T}\left(1-\left|{\hat{q}}_{t}\cdot q_{t}\right|\right)$$

where the dot product is given by ${\hat{q}}_{t}\cdot q_{t}=\hat{w}w+\hat{x}x+\hat{y}y+\hat{z}z$. This formulation ensures sign-invariant gradients and satisfies $L\left(q,\hat{q}\right)=L\left(q,-\hat{q}\right)$ and thus minimizes the angular misalignment directly.

During inference, orientation accuracy is evaluated using the geodesic distance on SO(3), a standard metric for sparse IMU-based motion capture systems [28]. Let the relative error quaternion be defined as $\Delta q_{t}={\hat{q}}_{t}⊗q_{t}^{-1}$. The angular error for a single joint at time *t* is formulated as

(25) $$E_{\mathrm{geo}}\left(t\right)=2\arccos\left(\mathrm{clamp}\left(\left|\Delta q_{t}\right|,\;-1.0,\;1.0\right)\right)\times \frac{180}{\pi }$$

The Mean Angular Error (MAE), reported in degrees, is calculated by averaging $E_{\mathrm{geo}}\left(t\right)$ over all joints and time steps.

The spatial accuracy of the reconstructed motion is evaluated using the Mean Per-Joint Position Error (MPJPE) after applying forward kinematics to the predicted joint rotations. For this purpose, a fixed skeletal model is assumed (e.g., humeral length ≈30 cm), similar to previous works [26]. Here, ${\hat{p}}_{t,j}$ and $p_{t,j}$ are used to represent the predicted and ground-truth 3D positions (in cm) of joint *j* at time *t* relative to the sternum root. The MPJPE is defined as

(26) $$\mathrm{MPJPE}=\frac{1}{T\cdot J}\sum_{t=1}^{T}\sum_{j=1}^{J}{∥{\hat{p}}_{t,j}-p_{t,j}∥}_{2}$$

This root-relative definition also explicitly accounts for the fidelity of structural pose. For instance, elbow flexion and shoulder abduction are quantified independently of global translation.

To complement spatial error metrics and distinguish between minor spatial deviations and severe pose failures, we also report the Percentage of Correct Keypoints (PCK), a widely used measure of robustness in human pose estimation [41]. PCK is defined as the proportion of joints whose Euclidean error falls below a specified threshold σ:

(27) $${\mathrm{PCK}}_{\sigma }=\frac{1}{T\cdot J}\sum_{t=1}^{T}\sum_{j=1}^{J}I\left({∥{\hat{p}}_{t,j}-p_{t,j}∥}_{2}<\sigma \right)$$

where I(·) denotes the indicator function. In this work, we report PCK@5 cm for strict precision and PCK@10 cm for general usability. Furthermore, we also report the PCK-AUC by integrating PCK over a range of thresholds from 0 to 15 cm. This provides a more general and threshold-independent measure of robustness.

Finally, to quantify the stability of the estimated pose over time and penalize high-frequency jitter in the perception, we also report the Temporal Smoothness Error (TSE). This metric measures deviations in second-order motion dynamics between the predicted and ground-truth trajectories, adapting approaches used to assess temporal plausibility in motion smoothing frameworks [42].

Let $p_{t,j}$ denote the 3D position of joint *j* at time *t*. We estimate physical acceleration $a_{t,j}$ using the discrete second derivative of joint position by scaling the finite positional difference by the squared time step $\Delta t^{2}$:

(28) $$a_{t,j}=\frac{p_{t+1,j}-2p_{t,j}+p_{t-1,j}}{\Delta t^{2}}$$

where Δt is the physical time duration between consecutive frames (i.e., Δt=1/60 s).

The Temporal Smoothness Error is then defined as

(29) $$E_{\mathrm{TSE}}=\frac{1}{(T-2)\cdot J}\sum_{t=2}^{T-1}\sum_{j=1}^{J}{∥{\hat{a}}_{t,j}-a_{t,j}∥}_{2}$$

Lower TSE values indicate greater temporal consistency and improved biomechanical plausibility, reflecting reduced high-frequency oscillations without over-smoothing dynamic motion.

### 6.4. Implementation Details

The proposed framework was implemented in PyTorch 2.1 and trained on NVIDIA GeForce RTX 4090 GPUs (24 GB VRAM). The 4.74M parameter model was parallelized across dual GPUs using *DataParallel*. The real-time inference bridge was implemented in Python 3.9 using the paho-mqtt library for low-latency communication, while visualization was performed in Unity 2022.3 LTS.

The network was trained using the AdamW optimizer with a fixed learning rate of 1 × 10^−4^ and batch size of 64. A fixed random seed of 42 was used for all network initializations and dataset shuffling operations to ensure reproducibility. To mitigate overfitting for the temporal window (*L* = 120), a dropout rate of 0.1 was applied across all Transformer encoder layers. The physics-informed multi-task loss was used with empirically tuned weights $\lambda_{q}=1.0$, $\lambda_{\omega }=0.1$, and $\lambda_{a}=0.01$ (See Equation (16)). The model was trained for 25 epochs with early stopping based on validation loss ($L_{MTL}$) with a patience of 10 epochs. The complete training procedure required approximately 14 h on the dual GPU configuration.

Consistent with the ablation study (Section 7.3), the final configuration uses a temporal context of L=120 frames (≈2.0 s) and a fixed hard negative mixing ratio of 10%, which translates to 10% hard negative samples and 90% natural ADL motion within each batch.

During real-time inference, the raw network predictions are processed using two lightweight post-processing steps (Section 5.4): (i) deterministic kinematic clamping to enforce joint limits and (ii) a manifold-aware multiplicative Kalman filter being applied to quaternion predictions (${\hat{q}}_{swing}$) to filter out jitter before streaming to the Unity avatar.

## 7. Results

### 7.1. Quantitative Performance Evaluation

The quantitative evaluation focuses on the system’s ability to sustain high fidelity in pose reconstruction for both daily activities and sophisticated joint isolation tests and is split into overall performance evaluation and model learning dynamics evaluation.

#### 7.1.1. Overall Sparse Reconstruction Fidelity and Error Distribution Analysis

**Figure 8:**
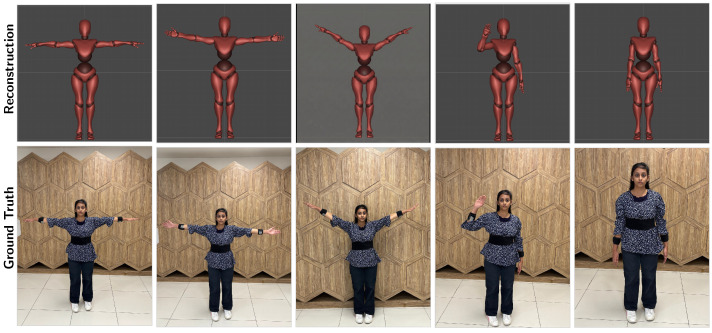
Qualitative comparison of reconstructed poses against ground truth under sparse sensing. The top row represents reconstructed avatar poses and the bottom row represents ground truth images. The images cover symmetric extension of arms, asymmetric motion of arms, and static poses. The model is capable of reconstructing joint positions and poses for different configurations of motion.

Table 2 lists the evaluation metrics and detailed statistical variance analysis of the proposed framework. The model attains a local MPJPE of 5.96 cm, well within the tolerable range (≈10 cm) for avatar control and teleoperation tasks. Moreover, a PCK-AUC of 0.733 indicates that the system sustains reconstruction accuracy for different motion velocities, addressing the problem of unobservability. The precise joint alignment and accurate spatial reconstruction for different kinematic configurations are qualitatively visualized in Figure 8. In order to assess robustness for arbitrary motion, standard deviations ($\sigma_{frame}$) are reported for the test set exclusively. This is because we want to ensure accurate evaluation of out-of-distribution generalization for unconstrained motion.

The reporting methodology along with its error distribution results provides essential insights about both biomechanical functions and statistical methods.

Statistical Reporting Methodology: The report has the standard deviation values (σ) for continuous spatial and temporal variables only (MAE, MPJPE, TSE) to show how sample values spread out from the center. The Percentage of Correct Keypoints (PCK) metric does not include standard deviation values (σ) according to its measurement standards. The binomial proportion (*p*) shows variance through its mean value, which follows the equation (p(1−p)), thus making empirical standard deviation calculations unnecessary because they do not show anything about the distribution except for what the mean value shows.

Frame-Wise Aggregation over IID Assumptions: The mean spatial error (MPJPE) results from calculating the average between all joint-frame pairs, which results in matching frame-wise mean values. The standard deviation measures only through frame-wise calculations, which lead to frame-wise results ($\sigma_{frame}$). Human skeletons operate as interconnected kinematic systems, so joint errors at time *t* show a different distribution pattern than Independent and Identically Distributed (IID) errors. The calculation of variance across joint-frame pairs that function independently would create an incorrect increase in denominator values, which would show a flawed representation of how errors actually spread through the system. The frame-wise standard deviation measures all temporary changes that occur during the process of pose degradation throughout time.

Distributional Response to Hard Negatives: The Coefficient of Variation (CV=σ/μ) reveals distinct, physically grounded distributions across the evaluation metrics. Geometric and spatial accuracy exhibit moderately elevated CVs (≈0.43 for MAE, ≈0.51 for MPJPE), while the temporal smoothness error (TSE) exhibits a high CV of 0.95. While standard literature often reports tighter variances by evaluating on smooth, highly predictable datasets (e.g., standard walking cycles), these elevated variances are an expected result of explicit hard negative mining. The model is capable of processing extreme, unconstrained transients due to the use of a robust ±5σ feature standardization during feature extraction, which vastly outperforms naive min-max scaling in preserving high-kurtosis signal spikes. The spatial CVs (≈0.5) prove graceful degradation of the model within a right-skewed, bounded distribution. More importantly, the TSE CV (≈0.95) proves that it is capable of suppressing micro jitter at the baseline (keeping the mean low) while allowing valid, high acceleration impacts to pass through the kinematic chain without acting as an overly aggressive low-pass filter, thus accurately mimicking natural human reflex.

Synergistic Spatial Compensation: Finally, analyzing the geometric relationship between angular and spatial distributions reveals the efficacy of the Transformer’s kinematic modeling. For a standard rigid human limb (e.g., humeral length L≈30 cm), a naive Forward Kinematics (FK) propagation of the mean angular error (15.30°) yields an expected spatial displacement error of L·sin(MAE)≈30·sin(15.30°)≈7.91 cm. The model’s actual local MPJPE is 5.96 cm. The mathematical reality that MPJPE <L·sin(MAE) empirically demonstrates that the network does not blindly accumulate rigid angular errors along the kinematic chain; instead, it learns synergistic, multi-joint spatial compensations that actively minimize the final Euclidean distance.

#### 7.1.2. Learning Dynamics & Convergence

**Figure 9:**
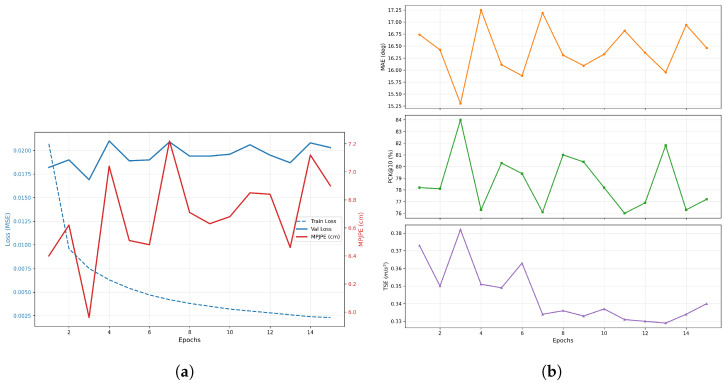
Learning dynamics of the proposed Transformer approach. (**a**) The total loss and local MPJPE metrics show fast convergence and stable performance through their complete evolution across epochs. (**b**) The progression of Mean Angular Error (MAE), PCK@10 scores and TSE demonstrates that the system achieves precise spatial accuracy while maintaining smooth trajectory movement.

Figure 9a shows the training stability of the physics-informed objective. The training and validation losses reach their convergence point after 10 epochs, which demonstrates that the $L_{MTL}$ objective successfully maintains regularization over the latent space without causing overspecialization. Figure 9b demonstrates the established connection between geometric precision and the temporal continuity that was learned by the system. As the spatial fidelity (PCK@10) increases and angular error (MAE) minimizes, the Temporal Smoothness Error (TSE) concurrently stabilizes at 0.316 m/s^2^. This proves that the model actively learns biomechanically continuous trajectories, rather than exploiting erratic, high-frequency target jumps to artificially minimize Euclidean distance.

#### 7.1.3. PCK Sensitivity Analysis

**Figure 10:**
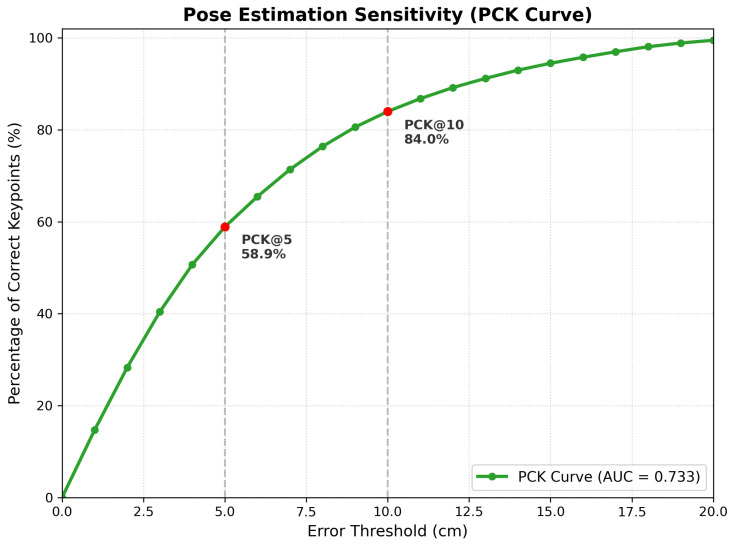
The PCK Analysis measures the Percentage of Correct Keypoints in relation to different Error Thresholds. The curve shows high sensitivity because it climbs steeply to show that most errors are mainly minor local deviations that do not result in kinematic failures.

The evaluation of the Percentage of Correct Keypoints (PCK) over a range of positional error thresholds in Figure 10 also follows a continuous spectrum. Although the evaluation of the PCK at a discrete set of thresholds (e.g., PCK@10) is a discrete evaluation that yields a binomial distribution, the evaluation over all thresholds effectively yields the Empirical Cumulative Distribution Function (eCDF) for the spatial errors. The sharp rise in the Empirical Cumulative Distribution Function also mathematically validates that the majority of the errors take the form of local positional deviations that are less than 5 cm in magnitude rather than any sort of kinematic failure mode. The ability of the model to locate 84.0% of the joint positions within a 10 cm positional error window also validates that the root-relative reconstruction approach is still valid even in the case of hard negative motion examples where the joint latency is high.

#### 7.1.4. Continuous Time-Series Validation

**Figure 11:**
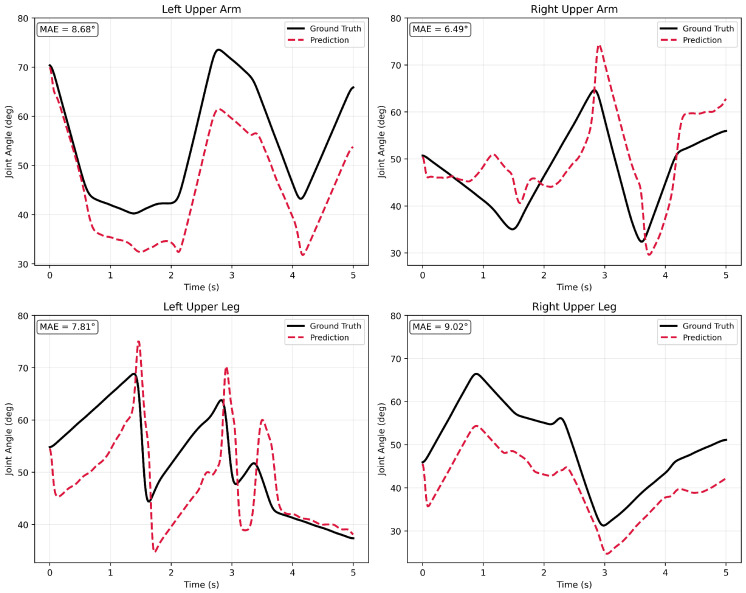
Continuous time-series validation of proximal joint stability during dynamic motion. The panels depict a 5-s window comparing ground-truth angular trajectories (solid black) against the Transformer predictions (dashed red) for the four uninstrumented proximal joints.

To visually substantiate the temporal stability and high-frequency noise suppression capabilities of the proposed architecture, we conducted a continuous 1D time-series analysis. Figure 11 illustrates a 5-s inference window extracted from the evaluation set, tracking the angular trajectories of four primary uninstrumented proximal joints (left and right upper arms, left and right upper legs) during a highly dynamic sequence.

The plotted trajectories correspond to a complex, multi-limb physical interaction involving asynchronous reaching, stabilization, and weight-transfer phases under dynamic perturbation. In the upper body, the left upper arm exhibits a distinct sequence of controlled lowering, brief stabilization, and a sharp overhead reach. Concurrently, the right upper arm demonstrates rapid compensatory stabilization (characterized by sharp, bounded dips) in response to high-frequency distal activity at the wrist. In the lower body, the trajectories capture the biomechanics of balance and locomotion; the left upper leg reflects the stabilization phase of a forward step, while the right upper leg exhibits controlled flexion during a load-bearing stance.

Crucially, the Transformer network successfully reconstructs these highly dynamic movements while suppressing Kinematic Bleed-Through (KBT). Despite the presence of rapid distal perturbations and complex multi-joint coordination, the proximal joint predictions remain temporally stable and physically consistent with the ground truth. The model accurately captures sharp, intentional directional transitions, such as the load-bearing drops and subsequent recoveries, without introducing high-frequency jitter. This provides direct visual confirmation of the architecture’s robust temporal continuity and its ability to decouple distal sensor chaos from proximal joint kinematics.

### 7.2. Comparative Analysis: Effect of Temporal Backbone

To strictly isolate the impact of the architectural backbone, all temporal networks under analysis were trained with an identical configuration: a temporal context of L=120 frames, the physics-informed multi-task loss $L_{\mathrm{MTL}}$, and a 10% hard negative mining ratio. The quantitative results of these ablation tests are presented in Table 3.

A comparative analysis of temporal backbones shows the distinct advantages of the Transformer’s global receptive field. While some of the recurrent architectures like LSTMs achieve a marginally lower spatial error (5.86 cm) due to their inherent low-pass filtering effect of recursive state updates, they struggle to preserve high-frequency motion fidelity. Conversely, the Transformer maintains comparable geometric accuracy while exhibiting superior retention of fast-moving distal joint dynamics. Furthermore, the Transformer demonstrates superior performance over the Temporal Convolutional Network (TCN), achieving a 0.3 cm reduction in MPJPE alongside higher PCK@10 scores. This increased spatial tolerance also validates that global self-attention mechanisms that correlate preparatory motion with instantaneous joint movement are far superior to the limitations imposed by dilated convolution for tracking motion over a longer time period.

Notably, all the temporal backbones under evaluation retain sub-7 cm MPJPE, thereby verifying that the proposed sparse sensing framework is architecture-agnostic. The major reconstruction benefits are obtained with the proposed physics-informed objective function and hard negative mining strategy; however, the Transformer is determined to be the best temporal aggregator due to its ability to retain both spatial precision and temporal accuracy with the capability for extremely stable training with long context lengths (L=120), thereby avoiding any gradient degradation problems that may arise with RNNs.

### 7.3. Ablation Study

#### 7.3.1. Component-Wise System Ablation

Table 4 provides a detailed ablation study for the proposed sparse sensing framework with respect to the synergistic contribution of the proposed physics loss components ($L_{vel}$, $L_{acc}$), hard negative mining strategy, and post-inference constraints. In order to rigorously test the loss dynamics, a constant 10% hard negative ratio is employed for all configurations.

A component-wise analysis of our method reveals the synergy of our proposed system. Removing only hard negative mining degrades spatial accuracy (MPJPE of 6.47 cm compared to 5.96 cm of our baseline). Indeed, if we train on steady motions of ADLs only, the model fails to capture the motion dynamics of high-frequency isolation tasks. This verifies our claim that hard negative mining is required to expose our model to difficult edge cases and avoid kinematic bleed-through.

The multi-task incorporation of $L_{vel}$ and $L_{acc}$ is critical to ensure biomechanical realism and spatial accuracy. The network becomes highly vulnerable to the 10% hard negative isolation tasks when the inertial gradients are eliminated, resulting in a highly divergent spatial and temporal result (MPJPE spikes to 9.78 cm and TSE = 1.476 m/s^2^).

In the inference step, only excluding kinematic constraints leads to a moderate level of spatial degradation (MPJPE at 6.68 cm) with significant levels of temporal jitter (TSE equals 0.720 m/s^2^). The significant impact of zero-mean jitter at higher frequencies on the second-derivative-based TSE leads to the unconstrained boundaries causing the joints to move outside the tolerance zone of 5 cm, thereby causing a significant drop in PCK@5 from 58.9% to 54.2%.

Finally, the Vanilla baseline, which is a standard transformer network trained only on geometric orientation loss $L_{quat}$, points out an essential optimization paradox. While the Vanilla baseline has a smaller spatial error (7.39 cm) than the *w/o Physics Loss* counterpart by construction, merely by avoiding the hard negative data points, the Vanilla baseline can easily overfit the mean ADL poses. Nevertheless, the unconstrained optimization on the Vanilla baseline yields an extremely high perceptual jitter (2.421 m/s^2^), confirming the importance of physics continuity for avoiding a catastrophic failure in the temporal domain.

#### 7.3.2. Hyperparameter Tuning of the Multi-Task Loss

In order to rigorously evaluate the impact of each kinematic penalty within the physics-informed Multi-Task Learning (MTL) loss function, a comprehensive ablation study over the entire range of the velocity ($\lambda_{\omega }$) and acceleration ($\lambda_{a}$) weights was carried out, keeping the geometric orientation weight ($\lambda_{q}$) fixed at 1.0. In contrast to the component ablation study above, the entire architectural pipeline, including the 10% hard negative injection and the post-inference constraints, was kept entirely enabled for this evaluation. This guarantees that the evaluation, which is carried out strictly on the validation set, is designed to isolate the gradient dynamics of the relative loss scalings without overfitting the final test distribution. The results are summarized in Table 5.

Significantly, the metric progression through the various hyperparameter configurations demonstrates a clear progression from an under-constrained manifold to an over-constrained rigid state. The baseline *Quaternion Only* configuration shows a severe geometric misalignment (MAE = 20.84°) and a high spatial divergence (MPJPE = 9.78 cm), coupled with a low PCK@10 (57.3%) indicating a high tracking failure rate. Adding a first-order penalty term to the loss function under the *Velocity Only* configuration significantly improves the angular accuracy (18.06°), reducing the temporal jitter by half; however, a high TSE of 0.853 m/s^2^ still fails to provide a perceptually stable solution, empirically verifying the inability of first-order constraints to address the high-frequency ambiguities present.

On the other hand, an overzealous application of the kinematic penalties artificially introduces stiffness. In the *Acceleration Over-Damped* configuration ($\lambda_{a}=0.5$), an overzealous smoothing effect is enforced to maintain a constant TSE (0.325 m/s^2^). However, this artificially imposed stiffness actively damps valid high-frequency motion transients and hence compromises the spatial accuracy (MPJPE = 6.84 cm) and robustness (PCK@5 drops to 55.1%). This gradient clash is particularly pronounced for the *Rigidly Constrained* configuration ($\lambda_{\omega }=0.5,\lambda_{a}=0.5$). In the pursuit to minimize the physical velocity at the cost of the correct joint positioning, the network induces severe angular misalignment (MAE soars to 18.94°), spatial mode collapse (MPJPE = 7.52 cm), and a drastic loss in robustness (PCK@10 drops to 74.8%).

The proposed *Critically Damped* configuration, i.e., $\lambda_{\omega }=0.1$ and $\lambda_{a}=0.01$, effectively grounds the optimization landscape, yielding the global optimum for both geometric error (MAE = 15.30°) and spatial displacement (MPJPE = 5.96 cm), as well as maximizing the robustness of the tracking solution over the entire dataset (PCK@10 = 84.0%). Most importantly, this configuration serves as a balanced form of regularization, which effectively maintains the natural dynamics of motion (TSE = 0.316 m/s^2^), without succumbing to over-constriction of the model. This observation again supports the notion that moderate inertial regularization is beneficial for achieving increased accuracy in both geometric and spatial reconstructions, whereas over-regularization causes the optimization process to converge towards an artificially smooth motion at the expense of pose fidelity.

#### 7.3.3. Sensitivity to Hard Negative Injection Ratio

To determine the optimal balance between the modeling of typical ADLs and of challenging isolation tasks, the network’s spatial and temporal performance was evaluated across varying hard negative mix ratios, as shown in Table 6.

The metric progression shows 10% as the optimal trade-off operating point between isolation robustness and natural motion smoothness. Omitting hard negatives entirely (0%) yields suboptimal isolation robustness, producing an MPJPE greater than 6.4 cm. Injecting 10% hard negatives achieves the global minimum spatial error (5.96 cm) by providing sufficient gradient signal to learn the noise-suppression decision boundary without disrupting natural synergistic motion priors. However, over-saturating the batch with chaotic motion (>10%) causes the model to become overly conservative. In these cases, the network mistakes valid rapid ADL transients for noise, which degrades general spatial performance and tracking reliability.

#### 7.3.4. Impact of Temporal Context Length (*L*)

**Figure 12:**
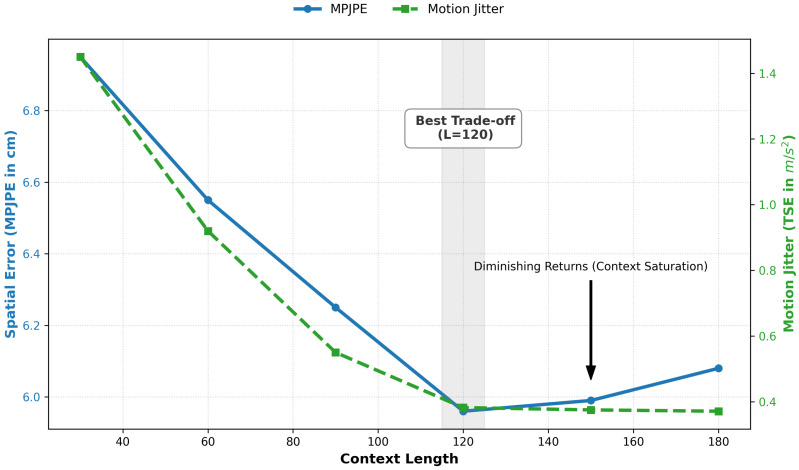
Visualizing the receptive field trade-off. The local MPJPE demonstrates a clear convex “U-shaped” profile as historical context increases.

We analyzed the effect of the input window size on overall reconstruction fidelity, with the quantitative and visual trade-offs illustrated in Table 7 and Figure 12.

The input window size heavily dictates reconstruction fidelity, exhibiting the established convex trade-off profile. Short context windows (L<60) fail to capture the low-frequency preparatory phase of motion, leading to response-based prediction lag. On the contrary, overly large window sizes (L>120) cause distracting “historical noise” for the attention mechanism, thereby causing spatial performance degradation. An optimal temporal context of L=120 frames (approximately 2.0 s) effectively aggregates sufficient history to handle motion duality while minimizing both geometric error and spectral smoothness.

### 7.4. Real-Time Performance Analysis

**Figure 13:**
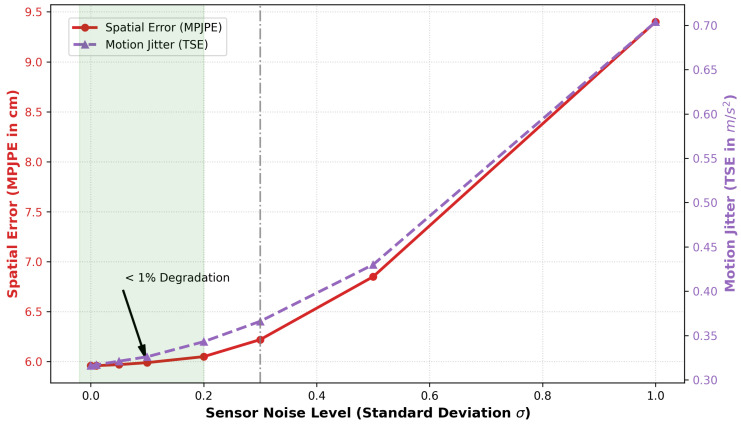
Impact of Sensor Noise on Reconstruction Fidelity. The MPJPE degradation curve remains almost linear for σ≤0.3, demonstrating the denoising capacity of the Transformer. A sharp raise at σ=0.5 marks the critical failure threshold for signal separation.

During actual implementation, IMU signals face disruption from ferromagnetic interference along with MEMS thermal drift and transmission packet loss. The system’s reliability under extreme conditions was assessed through a stress test which involved adding Gaussian noise with distribution $N(0,\sigma^{2})$ to the input accelerometer and gyroscope channels. Table 8 and Figure 13 illustrate the quantitative and visual degradation of reconstruction fidelity relative to this noise intensity (σ).

The system’s reliability under extreme conditions exhibits a non-linear response to sensor noise, characterized by three distinct stability zones. In the *Invariance Zone* (σ≤0.1), the physics-informed priors successfully remove micro-jitter, allowing the model to maintain high stability with only a 0.5% increase in MPJPE. As noise increases into the *Stability Zone* (σ≤0.3), degradation remains acceptable (+4.3%), as the physics loss components ($L_{vel}$, $L_{acc}$) successfully bound the induced jitter to maintain a controlled TSE score of 0.366 m/s^2^. However, a critical *Failure Zone* emerges at σ≥0.5. At this threshold, the signal-to-noise ratio drops below the capacity of the self-attention mechanism to distinguish between genuine hard negative tremors and random channel noise, resulting in a sharp 14.9% increase in spatial error and the appearance of visually perceptible trajectory artifacts.

### 7.5. Comparative Benchmarking Against State-of-the-Art

To ensure a methodologically sound comparison, benchmarking is strictly restricted to state-of-the-art sparse reconstruction methods that report local/root-relative error, thereby decoupling articulation accuracy from the global translation drift inherent to inertial dead-reckoning. Table 9 compares the proposed framework against the optimization-based Sparse Inertial Poser (SIP) [28], the learning-based Deep Inertial Poser (DIP) [43], and the TransPose framework [44]. EM-Pose [45] is included solely as a theoretical upper bound, as it relies on drift-free electromagnetic trackers rather than IMUs.

The proposed method achieves a local MPJPE of 5.96 cm, outperforming the widely adopted DIP baseline (6.98 cm) [43] despite utilizing a reduced sensor configuration (5 IMUs versus 6). This result proves that long-horizon temporal aggregation (L=120) can successfully compensate for the spatial unobservability introduced by distal-only sparse sensing. While approaches such as TransPose achieve lower spatial error (4.90 cm) [44], they rely on larger sensor collections and non-causal techniques to access future video frames. The proposed framework, in turn, focuses on real-time applicability via a fixed causal window, providing an optimal trade-off between inference latency and accuracy for the control of interactive avatars. Furthermore, the accuracy of the proposed framework for the avatar position is superior to the state-of-the-art optimization-based baseline for this task, i.e., SIP (6.66 cm) [28], without the need for computationally expensive iterative inverse kinematics solvers.

## 8. Discussion

### 8.1. Results of Solving the Observability Problem via Hypothesis Space Reduction

As shown by the results, it is possible to achieve a high-precision local pose reconstruction with high accuracy (MPJPE = 5.96 cm) by only using five distal sensors. This is only possible by solving the physics observability problem via the use of temporal aggregation. This research shows that the reconstruction of human motion is not as simple as basic center point tracking; rather, more advanced methods are required. This is supported by the ablation study (Section 7.3), which shows a convex error curve with a maximum at L=120 frames. This effectively reduces the hypothesis space by utilizing two seconds of past information to disambiguate the dual motion problem where a single distal pose could theoretically map to multiple valid proximal configurations. This was necessary for the detection and mitigation of Kinematic Bleed-Through (KBT) issues.

Moreover, as discussed in the comparative analysis (Section 7.2), there is an important difference between raw geometric error diminishment and true biomechanical accuracy. Although recurrent baselines like LSTMs performed well in their capacity as low-pass filters to produce adequate mean error results, their performance was significantly diminished for fast and transient movements.

In contrast, our Transformer model demonstrated better high-frequency spectral accuracy (TSE ≈ 0.316 m/s^2^), thereby producing motion patterns that closely resembled actual ground truths. This was due to the attention mechanism successfully tracking relevant distal movements to assess proximal stabilities without overly smoothing the input.

However, there is an important need to establish the scope of these results. The accuracy of our results (<6 cm MPJPE) is very specifically local and root-relative. We do indeed obtain highly accurate internal kinematic chain articulation, even for complex movements like shoulder abduction and elbow flexion, precisely because we do not have to contend with global drift evaluation constraints.

### 8.2. Practical Implications

The proposed framework of sparse sensing is highly cost-effective and applicable for avatar creation and teleoperation, avoiding the need for optical trackers or full-body suits. As can be inferred from the noise ablation testing (Section 7.4), it is also evident that even when sensor noise is artificially introduced, it does not impede the system’s reconstruction capabilities, as the increase in MPJPE is under 5%. The proposed physics-informed transformer-based system is more robust than conventional drift-sensitive inertial reconstruction pipelines, which can experience significant drift accumulation when operating in magnetically polluted environments such as factories or cluttered indoor settings.

This “Sparse-to-Dense” system is highly applicable for desktop-driven telepresence and local avatar animation setups. By utilizing consistent distal joint kinematics, it can create a realistic avatar representation on standard consumer PC hardware without requiring precise body measurements or expensive optical arrays.

Besides entertainment and telepresence, the framework also presents promising results for remote rehabilitation and biomechanics. The ablation results concerning temporal smoothness (TSE ≈ 0.316 m/s^2^) prove the viability of the proposed framework. In telemedicine, it is as important to assess the quality of a patient’s movements as it is to detect any possible presence of tremor and fluidity of a patient’s range of motion. The proposed framework presents a robust framework for accurate evaluation of human motion, as it ensures biomechanical accuracy while handling transient movements such as rapid waves and punches without over-smoothing and constricting the mean pose.

### 8.3. Limitations

Nevertheless, the system has certain inherent limitations. The primary limitation of this system is the lack of absolute global positional tracking. Due to the double integration drift present in MEMS-based accelerometers, the system can only accurately compute the internal articulation (pose), but not the absolute translation. As a result, all the calculations were carried out within the Body-Centric Reference Frame (BCRF). This means the position of the avatar’s root needs to be either fixed or externally driven. This limits the current application of this system to only seated or stationary stances.

Moreover, the system relies heavily on statistical priors to operate effectively. Although the use of hard negative mining successfully overcame the Kinematic Bleed-Through issue for calibrated isolation tasks, the attention-based approach has certain inference limits for unpredictable extreme athletic performances. In this case, the system relies on a secure average pose prediction to prevent sudden spikes in invalid motion. This causes a temporary degradation in the expression of movement.

Finally, the noise ablation study points out the robustness threshold for the environment. While the system maintains functionality under standard sensor noise conditions, performance declines rapidly when noise exceeds σ=0.3. Therefore, deployment in heavy industrial environments with extreme ferromagnetic interference or severe mechanical vibration would require external magnetic calibration or superior IMU hardware, as these conditions exceed the operational ceiling of the current attention-based system framework.

## 9. Conclusions

The research developed a physics-informed sparse sensing system that can accurately reconstruct human movement patterns through a system that uses five remote IMUs. This study, which is confined to limb segments, is naturally extendable to the entire human body hierarchy system, with possible use cases including remote rehabilitation, training for sports, biomechanics, and computer graphics. The observability problem that exists because of restricted tracking capabilities is resolved by the system through its use of a Transformer combined with a multi-task physics objective to create a soft constraint system that distinguishes between distal joint jitter and actual proximal movement.

The temporal aggregation method, utilizing a context window of L=120 frames, and the hard negative mining strategy with a 10% injection ratio functioned as fundamental requirements that needed to be fulfilled before we could proceed with hypothesis space reduction and Kinematic Bleed-Through filtering. The analytical comparisons show that recurrent baselines operate as low-pass filters while the attention mechanism of our system preserves the high-frequency spectral fidelity that authentic avatar representations need to function properly.

The research establishes a connection between deep learning and biomechanics through its development of an advanced solution that operates with minimal resources while delivering stable performance during noise disturbances for virtual reality telepresence and remote rehabilitation systems.

## 10. Future Work

The existing local pose estimation system requires improvement through sensor fusion technology, which will establish global localization capabilities. The combination of sparse IMU data with headset-based Visual Odometry (VIO) and Extended Kalman Filter (EKF) will enable us to determine the avatar’s absolute world-space position because the inertial-only framework does not provide global translation estimation.

The performance results show that extreme noise conditions with σ>0.5 lead to performance degradation, so we must enhance system reliability for ferromagnetic environments as our upcoming development goal. Domain adversarial training allows future iterations to simulate extreme magnetic distortions, which will reduce dependence on magnetometers and provide accurate tracking capabilities in industrial environments with heavy magnetic interference.

The current system can deliver low-latency performance through discrete GPUs but requires specific optimization for use on standalone VR headsets that operate with Snapdragon XR2. The next research phase needs to examine post-training quantization (PTQ) for converting to INT8 formats, which will help decrease inference system requirements. Low-Rank Adaptation (LoRA) and QLoRA techniques offer effective methods for personalized device use because they enable users to customize their devices using their own personal data. The model requires only a small number of parameter adjustments to optimize itself for the user because the edge device handles unique biomechanics of the user through parameter changes that reduce the processing costs associated with full network retraining.

## Figures and Tables

**Table 1:** Technical Specifications of the Data Acquisition Hardware [33].

(a) Physical Properties			
Model	Dimensions	Weight	IP Rating
Xsens DOT (2nd Gen)	36.3×30.35×10.8 mm	11.2 g	IP68 (Water/Dust)
**(b) Sensor Specifications**			
**Module**	**Full Scale Range**	**Sensitivity** [34]	**Rate**
Accelerometer	±16 g	2048 LSB/g	800 Hz
Gyroscope	±2000°/s	16.4 LSB/°/s	800 Hz
Magnetometer	±8 Gauss	0.25 mGauss	60 Hz

**Table 2:** Overall evaluation metrics and statistical variance on the test set. The proposed method attains sub-6cm error in local space. Standard deviations ($\sigma_{frame}$) are reported for continuous variables. The model attains a healthy, bounded variance even when extreme kinematic anomalies are introduced.

Split	Loss (MTL)	MAE (°)	$\sigma_{\mathrm{MAE}}$ (°)	MPJPE (cm)	$\sigma_{\mathrm{MPJPE}}$ (cm)	PCK@5 (%)	PCK@10 (%)	TSE (m/s^2^)	$\sigma_{\mathrm{TSE}}$ (m/s^2^)	PCK-AUC
Training	0.0075	10.19	—	5.15	—	92.5	98.1	0.254	—	0.880
**Test**	**0.0169**	**15.30**	**6.56**	**5.96**	**3.06**	**58.9**	**84.0**	**0.316**	**0.301**	**0.733**

**Table 3:** Quantitative comparison of temporal backbones. Evaluated under identical training constraints, all architectures achieve competitive spatial accuracy, but the Transformer provides the optimal balance of geometric precision and high-frequency motion retention.

Model Architecture	MAE (°)	MPJPE (cm)	PCK@5 (%)	PCK@10 (%)	TSE (m/s^2^)
**Transformer (Ours)**	**15.30**	**5.96**	**58.9**	**84.0**	**0.316**
TCN	15.98	6.26	56.1	78.7	0.414
BiLSTM	15.28	6.20	62.0	79.4	0.332
GRU	16.17	5.99	59.0	83.9	0.373
LSTM	15.302	5.86	62.0	85.1	0.325
RNN	16.03	6.15	51.8	84.1	0.365

**Table 4:** Component-wise ablation of the proposed sparse sensing framework. The configurations isolate the synergistic contributions of data augmentation, the physics-informed multi-task objective, and post-inference filtering.

Component	MAE (°)	MPJPE (cm)	PCK@5 (%)	PCK@10 (%)	TSE (m/s^2^)
**Ours (Full System)**	**15.30**	**5.96**	**58.9**	**84.0**	**0.316**
w/o Hard Negative Mining	17.54	6.47	51.1	75.6	0.333
w/o Physics Loss Components	20.84	9.78	33.8	57.3	1.476
w/o Post-Inference Constraints	16.55	6.68	54.2	77.1	0.720
Baseline (Vanilla)	17.78	7.39	53.4	73.2	2.421

**Table 5:** Hyperparameter Ablation of the Physics-Informed Multi-Task Loss. Quantitative trade-offs of inertial parameters regularization scaling. The proposed critically damped configuration ($\lambda_{q}=1.0,\lambda_{\omega }=0.1,\lambda_{a}=0.01$) achieves an optimal balance by strictly minimizing spatial & angular alignment errors while maintaining biomechanical continuity.

Configuration	$\lambda_{q}$	$\lambda_{\omega }$	$\lambda_{a}$	MAE (°)	MPJPE (cm)	PCK@5 (%)	PCK@10 (%)	TSE (m/s^2^)
Quaternion Only	1.0	0.0	0.0	20.84	9.78	33.8	57.3	1.476
Velocity Only	1.0	0.1	0.0	18.06	6.65	54.2	75.1	0.853
Under-Damped	1.0	0.01	0.01	16.85	6.38	56.7	76.7	0.482
**Critically Damped (Proposed)**	**1.0**	**0.1**	**0.01**	**15.30**	**5.96**	**58.9**	**84.0**	**0.316**
Velocity Over-Damped	1.0	0.5	0.01	16.27	6.47	50.3	79.3	0.405
Acceleration Over-Damped	1.0	0.1	0.5	16.64	6.84	55.1	79.4	0.325
Rigidly Constrained	1.0	0.5	0.5	18.94	7.52	50.7	74.8	0.331

**Table 6:** Impact of hard negative mix ratio. Quantitative trade-offs of data augmentation scaling.

Mixing Ratio (*r*)	MAE (°)	MPJPE (cm)	PCK@5 (%)	PCK@10 (%)	TSE (m/s^2^)
0.0 (Baseline)	17.54	6.47	51.1	75.6	0.333
**0.1 (10%)**	**15.30**	**5.96**	**58.9**	**84.0**	**0.316**
0.2 (20%)	15.62	5.98	55.8	80.4	0.407
0.3 (30%)	16.01	6.58	54.7	80.2	0.378
0.4 (40%)	15.24	6.05	58.0	83.2	0.392
0.5 (50%)	15.94	6.28	55.4	82.3	0.463

**Table 7:** Quantitative impact of temporal context length (*L*) on reconstruction fidelity across geometric and temporal metrics.

L (Frames)	MAE (°)	MPJPE (cm)	PCK@5 (%)	PCK@10 (%)	TSE (m/s^2^)
30 (0.5s)	17.50	6.95	48.0	75.0	1.450
60 (1.0s)	16.80	6.55	52.0	78.5	0.920
90 (1.5s)	16.20	6.25	55.0	81.0	0.550
**120 (2.0s)**	**15.30**	**5.96**	**58.9**	**84.0**	**0.316**
150 (2.5s)	15.305	5.99	58.5	83.8	0.375
180 (3.0s)	15.48	6.08	58.0	83.2	0.371

**Table 8:** Quantitative Stress Test. Breakdown of error metrics across noise intensities. System fidelity remains stable (<6.3 cm) within the standard MEMS operational range (σ≤0.3).

Noise Level (σ)	MPJPE (cm)	TSE (m/s^2^)	Degradation (%)
**0.00 (Clean)**	**5.96**	**0.316**	**0.0%**
0.01	5.96	0.317	0.0%
0.05	5.97	0.321	+0.1%
0.10	5.99	0.326	+0.5%
0.20	6.05	0.343	+1.5%
0.30	6.22	0.366	+4.3%
0.50	6.85	0.430	+14.9%
1.00 (Noisy)	9.40	0.704	+57.7%

**Table 9:** Comparison with state-of-the-art sparse reconstruction methods. All metrics report local/root-relative MPJPE (excluding global drift) to ensure a standardized baseline.

Method	Year	Sensors	Temporal Context	Local MPJPE (cm)	Local MAE (°)
SIP [28]	2017	6 IMU	Optimization	6.66	8.77
DIP (BiRNN) [43]	2018	6 IMU	Short Window	6.98	14.41
TransPose [44]	2022	6 IMU	Long-Range/Non-Causal	**4.90**	**7.62**
EM-Pose [45] *	2021	6 EM	N/A	3.54	13.3
**Ours (Proposed)**	**2026**	**5 IMU**	**L=120 Frames**	**5.96**	15.30

* Note: EM-Pose establishes a drift-free theoretical maximum bound and cannot be directly compared to inertial tracking systems.

## Data Availability

The supplementary hard negative motion corpus and the associated data processing scripts developed for this study are publicly available as the SparseTrack Dataset v1.0 (https://github.com/suchirmv-1524/SparseTrack-Dataset, accessed on 17 May 2026) on GitHub. The Virginia Tech Natural Motion Dataset (VT-NMD) utilized for the primary Activities of Daily Living (ADL) motion corpus is available from its original authors [27].
